# Identification of a Genomic Reservoir for New *TRIM* Genes in Primate Genomes

**DOI:** 10.1371/journal.pgen.1002388

**Published:** 2011-12-01

**Authors:** Kyudong Han, Dianne I. Lou, Sara L. Sawyer

**Affiliations:** Section of Molecular Genetics and Microbiology, Institute for Cellular and Molecular Biology, The University of Texas at Austin, Austin, Texas, United States of America; University of Arizona, United States of America

## Abstract

Tripartite Motif (TRIM) ubiquitin ligases act in the innate immune response against viruses. One of the best characterized members of this family, TRIM5α, serves as a potent retroviral restriction factor with activity against HIV. Here, we characterize what are likely to be the youngest *TRIM* genes in the human genome. For instance, we have identified 11 *TRIM* genes that are specific to humans and African apes (chimpanzees, bonobos, and gorillas) and another 7 that are human-specific. Many of these young genes have never been described, and their identification brings the total number of known human *TRIM* genes to approximately 100. These genes were acquired through segmental duplications, most of which originated from a single locus on chromosome 11. Another polymorphic duplication of this locus has resulted in these genes being copy number variable within the human population, with a Han Chinese woman identified as having 12 additional copies of these *TRIM* genes compared to other individuals screened in this study. Recently, this locus was annotated as one of 34 “hotspot” regions that are also copy number variable in the genomes of chimpanzees and rhesus macaques. Most of the young *TRIM* genes originating from this locus are expressed, spliced, and contain signatures of positive natural selection in regions known to determine virus recognition in TRIM5α. However, we find that they do not restrict the same retroviruses as TRIM5α, consistent with the high degree of divergence observed in the regions that control target specificity. We propose that this recombinationally volatile locus serves as a reservoir from which new *TRIM* genes arise through segmental duplication, allowing primates to continually acquire new antiviral genes that can be selected to target new and evolving pathogens.

## Author Summary

A fundamental question in biology is how the immune system is able to inactivate the enormous number of pathogens that it faces. The vast majority of pathogens are quickly neutralized by the innate immune system, a large network of defenses to which approximately 1/30 of the human genome is devoted. Because pathogens are always evolving, these innate immunity genes must be able to acquire new specificities. Here we illustrate a novel mechanism of evolution that has been employed by the large family of *TRIM* innate immunity genes. We have found a cluster of tandemly arranged *TRIM* genes on chromosome 11 that serves as a “reservoir” from which new *TRIM* genes constantly arise. We show that this gene cluster is prone to spawning duplications of itself, allowing primate genomes to continually acquire new *TRIM* gene copies that can presumably be selected to combat present and new pathogens.

## Introduction

The TRIM protein family constitutes a newly appreciated group of innate immune effectors involved in the response to viral infection [1]–[3]. TRIM5α, one of the best studied members of this family, is a pattern-recognition receptor for mammalian retroviruses including HIV [4], [5]. TRIM5α assembles into a hexameric lattice on the surface of retroviral cores as they enter the cytoplasm of a newly infected cell [6]. This interaction stimulates premature capsid disassembly [7], [8] and the formation of unanchored K63-linked polyubiquitin chains that trigger the production of chemokines and cytokines including interferon [4], [9]. The *TRIM5* genetic locus has profound penetrance in determining viral titers in SIV (simian immunodeficiency virus) infected macaques [10]. *TRIM5* also serves as a significant genetic barrier to the transmission of retroviruses between primate species [5], [10]–[13]. Other TRIM proteins have been linked to infection by different families of viruses altogether. TRIM25 interacts with the influenza protein NS1 [14], [15] and also activates the inflammatory response through the production of unanchored K63-linked polyubiquitin chains [16]. TRIM23 interacts with human cytomegalovirus [17], TRIM56 with pestivirus [18], while TRIM19/PML confers resistance to a broad range of DNA and RNA viruses [19]. In fact, more than one third of the approximately 70 known human *TRIM* genes have been shown to be transcriptionally upregulated in response to interferons [20]. Although the mechanistic details behind how TRIM proteins perform their antiviral roles remain elusive in most cases, their profound relevance to viral infection is made clear by the many viral antagonists that have been shown to target them. For example, influenza, herpes simplex virus-1, human cytomegalovirus, and adenovirus are all known to encode proteins that interact with, or alter the activity of, human TRIM proteins [15], [17], [19].

**Figure 1:**
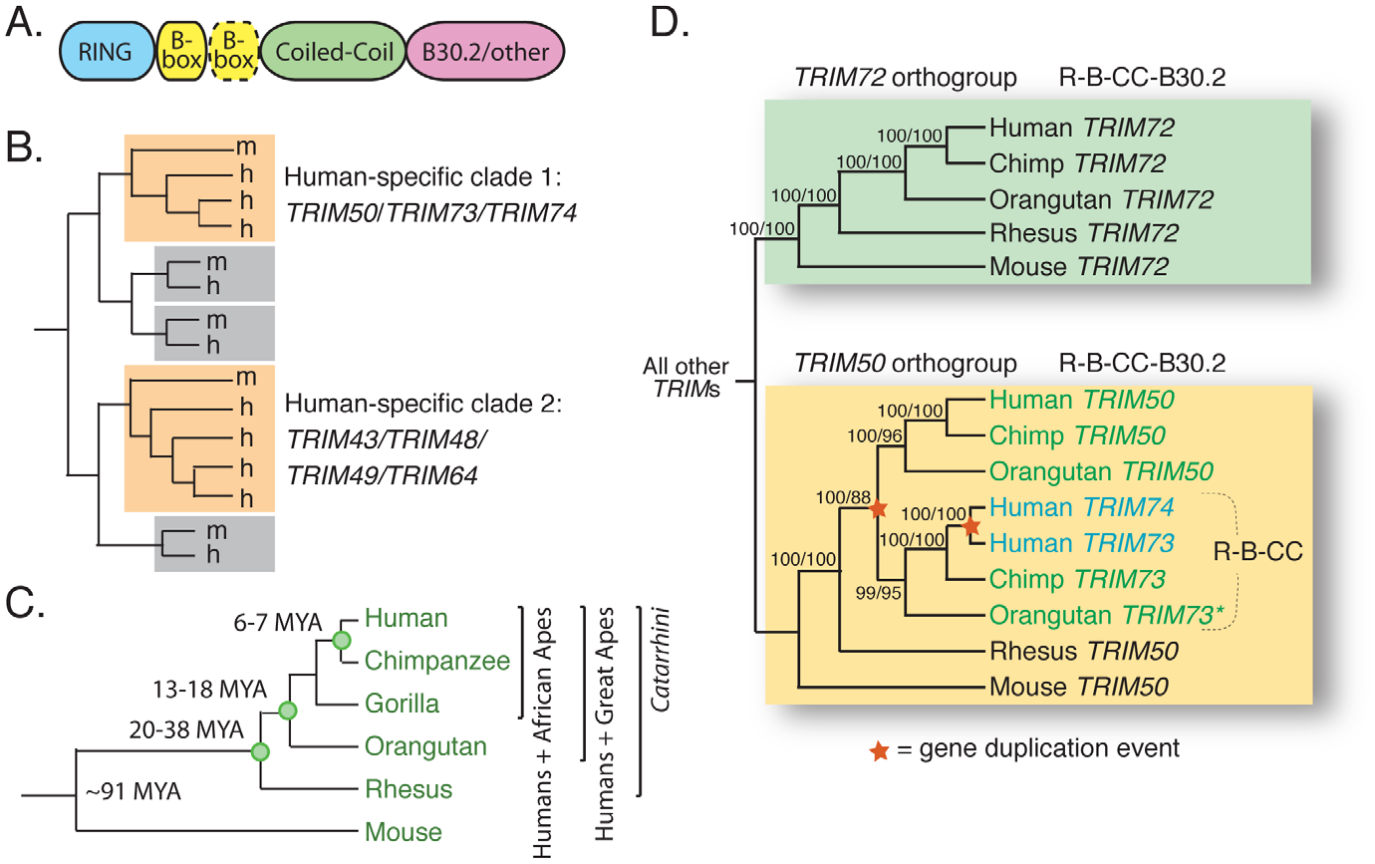
*TRIM73* and *TRIM74* arose in our recent primate ancestors. (A) A generic schematic of a TRIM protein is shown. There may be one or two B-boxes (yellow), and the final domain is variable although commonly a B30.2 domain. (B) An illustration summarizing the results of a previous analysis of human (h) and mouse (m) *TRIM* genes [2]. Most *TRIM* genes have strict 1∶1 orthologs between the two species, as illustrated by the pairs in gray boxes. Two clades of human-expanded *TRIM* genes were also noted (orange boxes). (C) The relationships of the species discussed in this study are shown, along with approximate dates of divergence [78], [79]. (D) A cladogram illustrates the relationship of *TRIM72/TRIM50/TRIM73/TRIM74* homologs present in the genomes of human, chimpanzee, orangutan, rhesus macaque, and mouse. The domain structure of the proteins encoded by these genes is indicated (R-B-CC is the tripartite motif discussed in the text). The asterisk (*) denotes orangutan *TRIM73*, which is an un-annotated gene located on an unassembled contig in the ponAbe2 genome assembly (7_random; positions 15,637,224–15,648,145). The tree was made from approximately 7,000 aligned bases in the R-B-CC region of these genes (introns and exons). Bootstrap values are shown for both neighbor joining and maximum likelihood methods (NJ/ML). A maximum parsimony tree was also constructed (not shown), and all nodes are supported by 88% or greater of bootstrap replicates regardless of the method used.

By definition, *TRIM* genes encode proteins with a conserved domain order: a RING zinc-coordinating domain, one or two zinc-coordinating B-boxes, followed by a coiled-coil domain (Figure 1A) [21]. These three domains constitute the “tripartite motif” that gives this family its name. Most *TRIM* genes also encode a variable C-terminal domain, and in many of them, this is a B30.2 domain [2]. The B30.2 is composed of a series of β-strands folded into a globular β-sandwich structure [22]. Different metazoan genomes contain different complements of *TRIM* genes. For example, *Drosophila melanogaster* has seven *TRIM* genes and *Caenorhabditis elegans* has eighteen [2]. In a previous comparison of the *TRIM* gene complements found in the human and mouse genomes, most were found to be strict 1∶1 orthologs [2]. This suggests that the majority of human *TRIM* genes are ancient, having originated more than 90 million years ago when human and mouse shared a last common ancestor. However, that study identified one phylogenetic clade of *TRIM* genes specific to the mouse genome, and two clades specific to the human genome. The clade of *TRIM* genes specific to the mouse genome (*TRIM12/TRIM30* and related genes) was subsequently shown to be an expanded set of *TRIM5* paralogs [23]. Based on this, we wished to describe the two phylogenetic clades of *TRIM* genes (*TRIM50/73/74* and *TRIM43/48/49/64*) which are specific to the human genome (Figure 1B). We also wished to determine whether these genes have been maintained by neutral drift or by selection, potentially imposed by evolutionarily recent viral infections.

In the process of characterizing these young human *TRIM* genes, we identified many additional, previously unidentified human *TRIM* genes to which they are closely related, bringing the total number of known human *TRIM* genes to approximately 100. We show that these novel genes have arisen from recent, and in some cases even human-specific, segmental duplication events. Specifically, we find that one locus on chromosome 11, containing nine tandemly situated *TRIM* genes, has spawned at least two separate segmental duplications of itself during the evolution of great apes, as well as having produced at least one other segmental duplication that is still polymorphic in the human population. This locus is therefore evolutionarily dynamic as well as copy number variable within the human population. In a fascinating example of trans-species copy number variation, this locus was recently annotated as one of 34 “hotspot” regions that are also copy number variable in the genomes of chimpanzees and rhesus macaques [24]. Trans-species copy number variation remains largely unstudied, and the evolutionary forces behind it remain unknown [25]. We propose that this locus is selected to remain recombinationally volatile so that it can serve as a reservoir from which new primate *TRIM* genes constantly arise. Theoretically, increased gene dosage of innate immunity genes, conveyed by increased copy number, could in itself provide protection against viral infection and disease progression. However, many of these genes are evolving under positive selection like other primate genes known to encode antiviral molecules [26]–[35]. Therefore, these duplicated genes also appear to be rapidly diversifying in function, possibly to expand the spectrum of antiviral affinities in response to new and evolving viruses.

## Results

### Young *TRIM* genes in the human genome

In a previous comparison of the *TRIM* genes found in the mouse and human genomes, several human-specific genes were noted (Figure 1B) [2]. Although these genes could have arisen anytime during the last 90 million years since human and mouse last shared a common ancestor, we were interested to know whether any of them have arisen during *Catarrhini* speciation (Figure 1C). This group constitutes our closest evolutionary kin, primates that have most likely faced pathogens similar to those that humans encounter. To address the evolutionary origins of these human-specific *TRIM* genes, we took advantage of the genome projects of several *Catarrhini* species, including chimpanzee and orangutan (both great apes), human, and rhesus macaque (an Old World monkey).

The first clade of human *TRIM* genes absent in the mouse genome contains *TRIM50*, *TRIM73*, and *TRIM74* (Figure 1B). To investigate when these genes arose, orthologous sequences were identified in the other *Catarrhini* genomes and a phylogeny was constructed (Figure 1D). The most closely related human outgroup sequence, *TRIM72*, was also included. *TRIM72* forms a clear orthogroup containing one gene from each species, with all nodes being consistent with speciation events (boxed in green). However, the *TRIM50/TRIM73/TRIM74* clade has been more dynamic (boxed in yellow). This branching pattern is consistent with an ancestral *TRIM50* that experienced two duplication events, each indicated by a star on the phylogeny. The first duplication, giving rise to *TRIM73*, occurred after the split between great apes and Old World monkeys, but before our last common ancestor with orangutan. It involved only the exons encoding the first three protein domains. The second duplication event occurred in the human lineage, less than 7 million years ago, giving rise to *TRIM74*. Consistent with two duplication events, *TRIM50*, *TRIM73*, and *TRIM74* reside near each other on three segmental duplications on human chromosome 7 [36]. Spliced transcripts have been identified for all three genes, and while TRIM50 has been demonstrated to act as an E3 ubiquitin ligase, the biological functions of TRIM73 and TRIM74 remain uncharacterized [36]. Thus, this small *TRIM* clade has gained gene copies though segmental duplications that have occurred during recent primate evolution. One gene, *TRIM74*, is even specific to humans.

**Figure 2:**
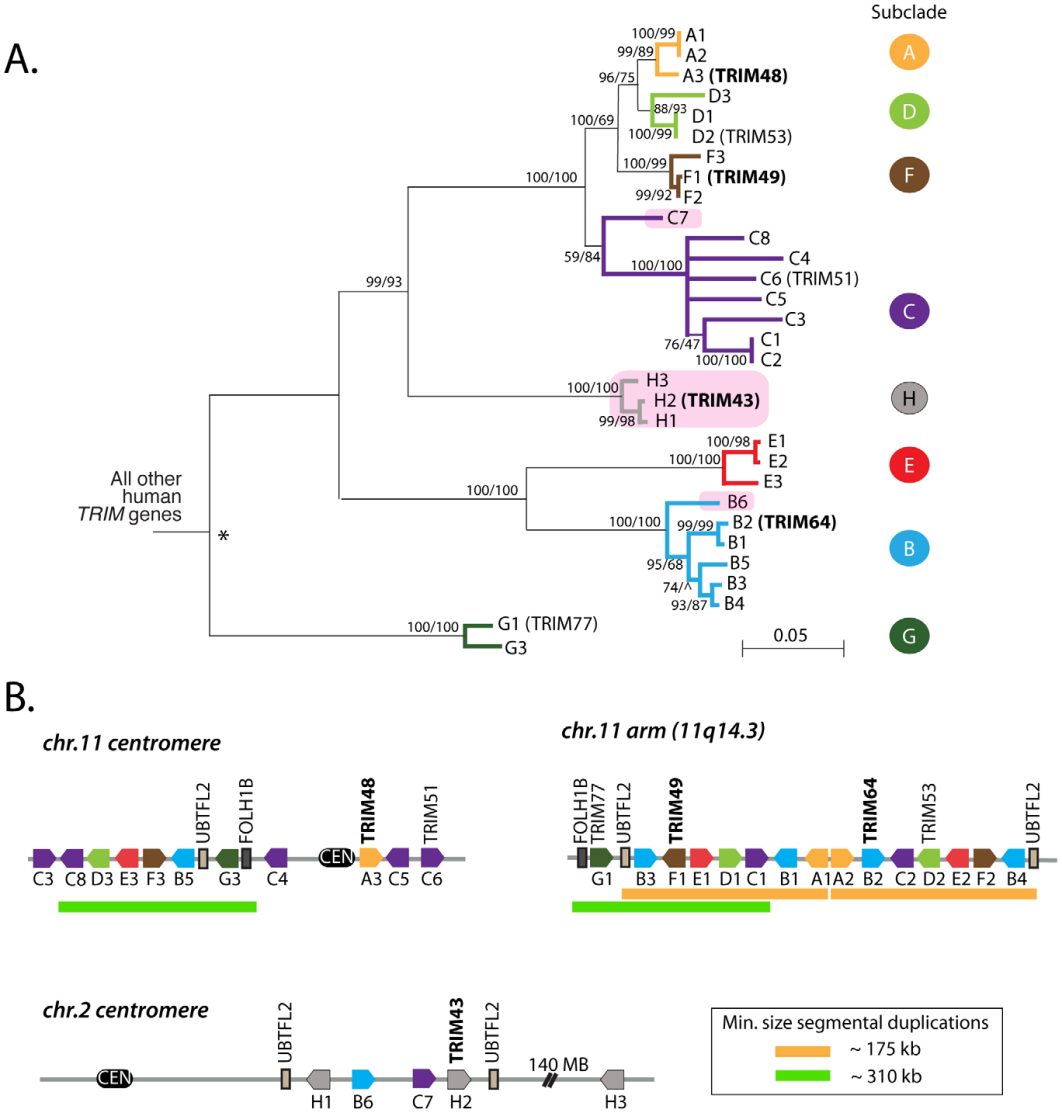
A dynamic clade of *TRIM* genes in the human genome. (A) 31 human paralogs were identified that group into a single phylogenetic clade. Seven of them have already been annotated with standard *TRIM* genes names (*TRIM48*, *TRIM51*, *TRIM77*, *TRIM49*, *TRIM53*, *TRIM64*, and *TRIM43*), including the four genes that were originally being investigated here (bold type). The rest are predicted genes that have been given temporary names reflecting their phylogenetic subclades (i.e. “A1”). Subclades of genes are color-coded for naming purposes. Pink boxes indicate *TRIM* genes located on chromosome 2, all other genes are on chromosome 11. The neighbor joining tree was based on an alignment of the predicted coding regions. Bootstrap values are shown for both neighbor joining and maximum likelihood methods (NJ/ML). Nodes are collapsed where support by both methods is <75%. The two methods yield different branching orders in only one case, in the B subclade at the node indicated (∧). (B) The genomic positions of these 31 *TRIM* genes are illustrated, according to the hg19 human genome assembly. Pentagons represent *TRIM* genes, with strand orientation designated by the direction of the symbol. The color of the gene symbol reflects the phylogenetic subclade to which the gene belongs (according to the tree in panel A). Green and yellow bars indicate two apparent inverted segmental duplication events. The segmental duplication at the chromosome 11 centromere actually spans the centromere [38], and is therefore likely to be substantially longer than the 310 kilobases indicated.

The second clade of human *TRIM* genes absent in the mouse genome contains *TRIM43*, *TRIM48*, *TRIM49*, and *TRIM64* (Figure 1B). Using reciprocal best hit analysis, we failed to detect strict 1∶1 orthologs between human and the other primates being analyzed. In fact, reciprocal searches of the human genome with primate orthologs continually returned large numbers of mostly un-annotated human genes. In all, we identified a group of 31 human *TRIM* genes that form a single monophyletic clade to the exclusion of all other *TRIM* genes in the human genome (Figure 2A). The clade includes seven *TRIM* genes previously assigned standard *TRIM* names (including the four used as queries, bold type) and 24 uncharacterized paralogs. Uncharacterized genes were given temporary names reflecting their phylogenetic subclade (i.e. A1 and A2 are two genes in the ‘A’ subclade shown in Figure 2A), but actual locus identifiers for each gene are given in Table 1. Of these 31 genes, 20 have full-length open reading frames that are predicted to encode tripartite motifs either with or without a C-terminal B30.2 domain (Figures S1, S2). Using RT-PCR on testes mRNA, we were able to identify processed transcripts for 11 of these 20 full-length genes, and when combined with cDNA reads available in Genbank, 14 out of 20 genes have evidence for expression and splicing (Figure S2). Thus, we have identified a large set of previously undiscovered human *TRIM* genes, most of which appear to have protein coding potential.

**Table 1:** Names and Refseq numbers for *TRIM* genes described in this study.

Temporary Name	Gene Name(s) Previously Assigned to this Locus	RefSeq Record
A1	LOC399939	NM_001206627.1
A2	TRIM49L1	NM_001105522.1
A3	TRIM48	NM_024114.3
B1	TRIM64B	NM_001164397
B2	TRIM64	NM_001136486
B5	TRIM64C	NM_001206631.1
C1	SPRYD5P	NG_011760
C2	LOC399940	NR_038146
C6	TRIM51/SPRYD5	NM_032681
C8	LOC120824	NM_001206625
D1	TRIM80P/TRIM53B	NG_012754
D2	TRIM53	NR_028346
D3	LOC340970	NG_021782
F1	TRIM49	NM_020358
F2	TRIM49L2	NM_001195234
F3	LOC283116	NM_001206626
G1	TRIM77	NM_001146162
G3	LOC646770	NG_028906
H1	TRIM43B	NM_001164464
H2	TRIM43	NM_138800
B3 (pseudo)	LOC727828	NG_028916.1
B4 (pseudo)	LOC440061	NG_028917.1
B6 (pseudo)	LOC390233	NG_028781.1
C3 (pseudo)	LOC100130105	NG_028918.1
C4 (pseudo)	TRIM49B	NG_028915.1
C5 (pseudo)	LOC440041	NR_038174.1
C7 (pseudo)	LOC643126	NG_028919.1
E1 (pseudo)	LOC642414	NG_028920.1
E2 (pseudo)	LOC642579	NG_028913.1
E3 (pseudo)	LOC100129108	NG_028914.1
H3 (pseudo)	LOC643445	NG_028773.1

The table shows information on how to retrieve DNA sequence for the genes discussed in this study. Genes towards the bottom of the table are predicted pseudogenes.

The 31 human *TRIM* genes in this clade are located at three genomic loci, one near the centromere of chromosome 2, one spanning the centromere of chromosome 11, and one on the arm of chromosome 11 at 11q14.3 (Figure 2B). When the genes in this schematic were color-coded to reflect the subclades in Figure 2A, it became clear that they arose through a series of segmental duplications. For example, the cluster of *TRIM* genes located on the chromosome 11 arm contains a mirror-image tandem inversion of 7 *TRIM* genes (denoted by two orange bars in Figure 2B). A second duplication event is evident in the region directly adjacent to the chromosome 11 centromere, where a stretch of 6 *TRIM* genes appears to be an inverted copy of part of the sequence located on the chromosome 11 arm (denoted by green bars in Figure 2B). The genes located near the chromosome 2 centromere cluster phylogenetically with those found on chromosome 11, suggesting that this may be yet another segmental copy, although the gene order is sufficiently degraded that we cannot draw any clear conclusions.

**Figure 3:**
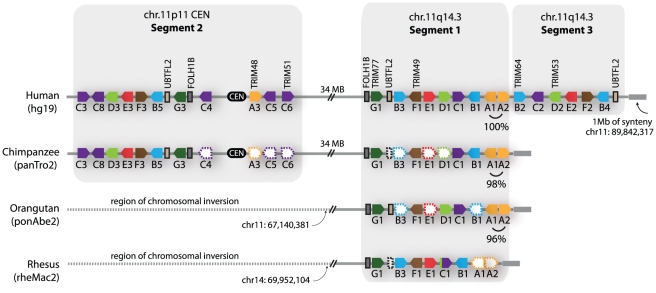
Primate comparative genomics reveals 11 African ape-specific and 6 human-specific *TRIM* genes. The chromosome 11 *TRIM* genes are diagrammed as they occur in the latest versions of four available primate genome projects. The dashed-empty pentagons represent genes that are presumed to be present, but could not be identified due to large regions of poor sequence quality in several of the genome projects. The dashed lines spanning the centromere in the orangutan and rhesus macaque genomes denote sequence that is not syntenic between genomes, due to a large rearrangement that has been described [37]. On the right-hand end of the diagrams, a 1 megabase block of synteny (grey bar) was identified that sits adjacent to segment 1 or segment 3 in all four genomes.

For discussion purposes, we denote the regions containing these apparent duplications on chromosome 11 as segment 1, segment 2, and segment 3, as illustrated in Figure 3. The duplicated chromosomal regions bearing these three segments are large, but we have focused only on the portion that contains *TRIM* genes. A careful inspection of the chimpanzee, orangutan, and rhesus macaque genomes in these regions was then performed (Figure 3). Segment 1 is found in all of these primate genomes, while segment 2 is found in the chimpanzee and human genomes only. In support of the young age of segment 2, segments 1 and 2 in the human genome are 96% identical along their length (calculated for 302 kilobase; Table S1). The identification of segment 2 in the genomes of orangutan and rhesus macaque is somewhat complicated by a large chromosomal inversion that has been reported in the region of the chromosome 11 centromere, which arose in the common ancestor of human, chimpanzee, and gorilla [37]. However, this segmental duplication has been previously analyzed by FISH, using BAC-derived probes that anneal in both segment 1 and segment 2 [37]. In that study, all primate species investigated showed a hybridization signal on the chromosome 11 arm at the location of segment 1. A second hybridization signal at the chromosome 11 centromere (segment 2) was observed in the genomes of human, chimpanzee, and gorilla, but not of orangutan, rhesus macaque, or any other primate tested, congruent with our conclusions made through comparative genomics. Therefore, segment 2 is a segmental duplication of segment 1 that is specific to humans, chimpanzees, gorillas, and presumably the final species of this monophyletic clade of African apes, bonobos. This is in agreement with a previous age estimate of 14 million years, based on sequence divergence, for the segmental duplication containing segment 2 [38].

Interestingly, segment 3 is found only in the human genome (Figure 3). The very recent acquisition of this segment as another copy of segment 1 is supported by the observation that segments 1 and 3 are 99.6% identical along their length (calculated for 170 kilobase; Table S1). In intra-chromosomal comparisons of all segmental duplications on chromosome 11, only 0.2 MB was found to have this level of identity [39], consistent with segment 3 being one of the newest segmental duplications on the entire chromosome. It is curious to note that genes A1 and A2, located in tail-to-tail fashion, are 100% identical along their length (over 6 kilobase) in the human genome, but only 96–98% identical in the genomes of orangutan or chimpanzee (Figure 3). A gene conversion event between A1 and A2 in the human genome may have accompanied or seeded the tandem inversion of segment 1 to create segment 3.

In summary, we have identified species- and human-specific *TRIM* genes on chromosome 11. The chromosomal region bearing segment 1 has been highly dynamic during the evolution of humans and African apes, seeding at least 2 segmental duplications in the last 18 million years since their last common ancestor with orangutan. There appears to be a gross dichotomy in the timing of *TRIM* gene acquisition by the human genome because, while the majority of human *TRIM* genes are ancient and arose >90 million years ago, the rest of them (approximately 20% of the *TRIM* genes in our genome) have arisen in very recent time, during the evolution of the great apes.

### Copy number variation

**Figure 4:**
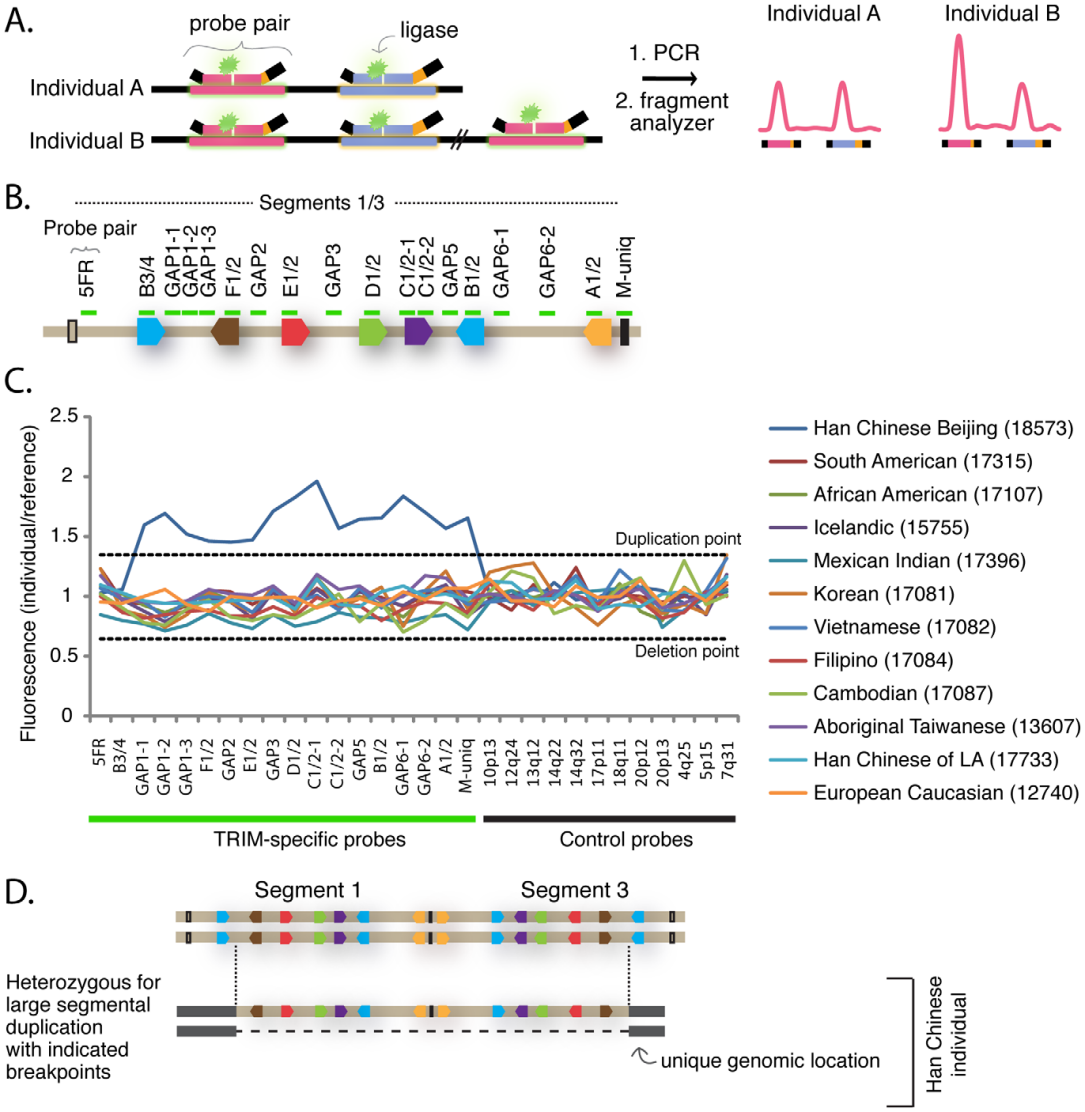
TRIM genes in segments 1 and 3 are copy number variable. (A) The schematic illustrates the Multiplex Ligation-dependent Probe Amplification (MLPA) assay. This assay utilizes pairs of probes designed to sit directly adjacent to one another at a particular genomic region. When the two probes anneal to the correct target region on denatured genomic DNA, the addition of a ligase results in their joining into a single, larger probe. Universal primer sites (black bars) at each end allow amplification of fragments from ligated pairs. Each probe pair yields a PCR product of unique length due to a “stuffer sequence” (orange) that is placed internally to one of the universal primer binding sites. The universal PCR primers are labeled with a fluorescent dye, and the quantity of each uniquely-sized fragment produced is measured with a fragment analyzer. (B) In this panel, ligated probe pairs are now illustrated with a single green bar. Eighteen probe pairs were designed to span segment 1, and are also a perfect match to their target sequence in segment 3. Thus, each is expected to anneal four times in a diploid genome. The one exception is the probe “M-uniq” which sits in a unique region between the segmental duplications. (C) The results of the MLPA assay are shown for 12 geographically diverse human samples. For each individual, the fragment intensity produced from each probe pair was normalized to the intensity produced by the Utah reference sample (NA10851). Control probes recognize single copy genes at the chromosomal locations indicated along the X-axis. For this reason, they yield the same quantity of fragments in experimental and reference genomes (all values hover between 0.65 and 1.35, the cut-offs for deletion and duplication). This is also true for the experimental probes. The one exception is in the genome of a Han Chinese sample (NA18573), where 16 adjacent experimental probe pairs (GAP1-1 through M-uniq) all yielded a quantity of fragments averaging 1.6× greater than the reference Utah genome. In all, 72 geographically-diverse human samples were assayed by MLPA, and a table of full results can be found in Table S5. (D) The MLPA results are consistent with a heterozygous duplication of most of the segment 1 – segment 3 locus.

Because the locus containing segment 1 has spawned multiple segmental duplications in recent primate history, we wished to determine whether there are also newer segmental duplications of this region that might be polymorphic in the human population. Genomic regions containing polymorphic segmental duplications or deletions greater than 1 kilobase in size are called ‘copy number variable’ (CNV) regions [40]. To characterize the population genetics of this locus, we employed the multiplex ligation-dependent probe amplification (MLPA) assay [41]. This is a PCR-based assay that utilizes a fragment analyzer to quantify the amount of product generated from different target regions in a genome (Figure 4A). Eighteen probe pairs were designed to tile across the length of segment 1 (Figure 4B and Table S4). Each probe in each pair anneals to 23–63 bases of genomic DNA, and is a perfect match to a target sequence in segment 1. Because of the high degree of similarity between segment 1 and segment 3, probes will also anneal to the cognate locus in segment 3 with perfect complementarily. However, probe pairs were carefully situated such that they have multiple mismatches to the corresponding sequence in segment 2, or to sequence anywhere else in the human genome (see materials and methods). Thus, each probe is expected to have four binding sites in a diploid genome because there will be two copies of each segment 1 and segment 3 target. The one exception is the probe pair “M-uniq,” which sits in a unique stretch of sequence between segments 1 and 3 and will have only two binding sites in a diploid genome. Control probe pairs that recognize standard single-copy genes distributed throughout the genome were also included (see materials and methods). Initially, 50 genomic DNA samples from individuals from around the world were analyzed. For each probe pair, the quantity of products produced from each sample was normalized to the quantity produced from a reference genome, as is standard for this assay. As the reference, we used the genome of a Caucasian male from Utah (NA10851) that has been previously used as the reference genome in several studies of CNV regions [40], [42]–[44].

The normalized fragment values for 12 representative genomes are graphed in Figure 4C, and values for all 50 surveyed genomes are presented in Table S5. We identified only one CNV, in the genome of a Han Chinese female (NA18573). In this individual, the 16 probe pairs spanning from GAP1-1 to M-uniq all yielded approximately 1.5 times the quantity of fragments as the reference genome (average enhancement across all 16 probe pairs = 1.6). This was verified in eight independent experiments (not shown). Therefore, this Han Chinese individual has two additional binding sites for each of these probe pairs, and one additional binding site for the M-uniq probe pair. This pattern is consistent with the segmental duplication scenario diagrammed in Figure 4D. A signal at this locus was previously detected in this same individual as part of a whole-genome array-based study that identified 1,447 human CNV regions [40]. Thus, we have identified a human individual of Chinese decent that has 43 *TRIM* genes belonging to this dynamic phylogenetic group instead of the 31 *TRIM* genes which most human individuals have.

Based on this finding, we then screened 22 additional Han Chinese samples, but did not find another instance of this segmental duplication (Table S5). In all, 72 human genomes were analyzed by MLPA (human samples are listed in Table S6). The polymorphism thus seems to be rare, as it was detected in only 1/72 human individuals. However, CNVs have also been detected at this locus, using array-based platforms, in the genomes of several other Asians, including 2 Japanese, 2 Koreans, and 1 additional Chinese individual (Figure S5) [40], [42], [44]. There is one report of a CNV at this locus in the genome of a Yoruban from Africa [43]. Perhaps most interestingly of all, the region containing segment 1 is also copy number variable in chimpanzees and rhesus macaques [24]. In summary, the region containing segment 1 has been highly dynamic both during primate speciation, and also in current human and primate populations.

### Positive selection of young *TRIM* genes

All of the findings described so far can potentially be explained as random events occurring in a dynamic genome. As segmental duplications arise, they may go to fixation through neutral drift even if there is no selection acting for or against them. A hallmark of genes that are being retained by neutral drift is that they accumulate equal rates of non-synonymous and synonymous mutations. Such genes have a characteristic signature of dN/dS = 1, where dN is the number of non-synonymous mutations per non-synonymous site, and dS is the number of synonymous mutations per synonymous site. In contrast, most functional genes accumulate non-synonymous mutations at a rate far slower than synonymous mutations (dN/dS<<1) due to the evolutionary constraint at play [45]. A third mode of evolution, recurrent positive selection, has influenced several *TRIM* genes in primate genomes, including *Pyrin/TRIM20* [46], *TRIM5* [28], [32], and *TRIM22* [27]. Genes or gene regions subject to such a selective regime accumulate a characteristic signature of dN/dS>1 [47].

We analyzed the evolutionary pressures that have shaped these young *TRIM* genes at the sequence level in order to determine whether they have been neutrally or selectively retained. Usually, evolutionary datasets of orthologous sequences are used for such analyses, but because these genes are so new and dynamic, deep species sets of strictly orthologous sequences cannot be easily obtained. Instead, we looked at the patterns of nucleotide substitution that have occurred during the diversification of these genes by comparing human paralogs, all of which can be traced to a common ancestral gene (asterisk in Figure 2A). Of the 31 *TRIM* genes in the dynamic clade being investigated, 15 are predicted to encode proteins with the full TRIM-B30.2 structure (Figure S2). However, of these, two very recently diverged gene pairs (A1/A2 and C1/C2) are still identical along the length of their coding sequence (Table S2), leaving 13 unique sequences which can be analyzed.

**Figure 5:**
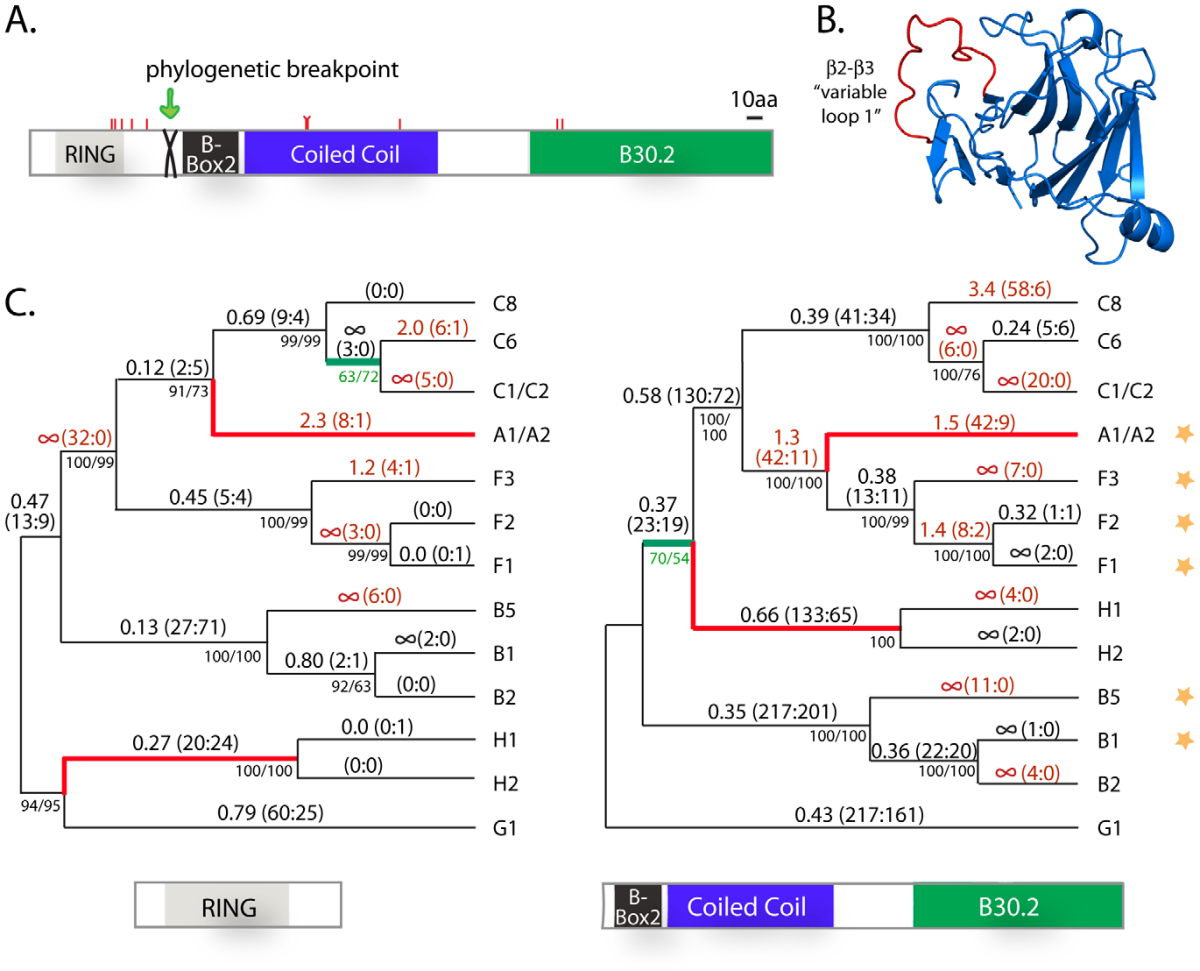
Positive selection has shaped the sequence of the novel *TRIM* genes on chromosomes 2 and 11. (A) A schematic where tick marks represent the 10 residue positions found to be evolving under positive selection in the novel TRIMs. Also indicated is a phylogenetic breakpoint which was detected in the sequence alignment, located between the RING and B-box 2 domains (after base position 237 in the coding sequence). (B) The crystal structure (PDB 3KB5) of a TRIM B30.2 domain is shown [80], with the β2-β3 loop highlighted in red. (C) The phylogenetic trees of the two halves of the alignment differ only in the placement of two branches (in red). Bootstrap support for each node is shown by both neighbor joining and maximum likelihood (NJ/ML) methods. On each branch, the estimated value of dN/dS is given, followed by the number of non-synonymous and synonymous mutations predicted to have occurred along that branch (N∶S). dN/dS is indicated as infinity where dS = 0. Text is highlighted in red where dN/dS >1 (or, arbitrarily, where N∶S≥3∶0 in cases where dS is zero). Genes A1 and A2, and genes C1 and C2, are identical along the length of their coding sequence. Stars on the right indicate genes that were functionally tested in retroviral restriction assays. Finally, each of the two trees has one poorly supported node (in green).

Importantly, analysis of sequence evolution requires an accurate phylogenetic representation of the genes being analyzed [48]. One problem with understanding the phylogenetic relationship of paralogs from a single genome is the fact that gene conversion may have occurred. To detect phylogenetic incongruencies in our alignment, indicative of such events, we used the GARD program [49] as described in the materials and methods. Only one phylogenetic breakpoint was identified (p<0.05), located between the RING and B-box2 encoding domains of the first protein-coding exon (Figure 5A). The alignment of the 13 *TRIM* genes was subsequently divided at this point and the trees produced by each half are shown in Figure 5C. Only two branches differ between the trees (highlighted in red), suggesting that gene conversion has not been extensive. The tree for each half is highly supported, regardless of the phylogenetic method utilized (Figure 5C), or whether just sites at the third positions of codons are utilized (data not shown). With these trees, each half of the multiple alignment was analyzed separately using the codeml package in PAML (see materials and methods). We analyzed each half of the alignment separately, under variable models of selection and codon usage (Table S3). All models yielded strong support for positive selection acting on both halves of the gene (p<0.05). Each of the two trees has one poorly supported node (highlighted in green; Figure 5C). We confirmed that support for positive selection remains strong when each of these nodes is collapsed (p<0.05; Table S3). Therefore, there is convincing evidence that these new *TRIM* genes have not been retained by neutral drift alone.

dN/dS values were calculated for each branch on the two trees. On these trees, there are many branches where dN/dS>1 (text highlighted red; Figure 5C). In fact, the dN/dS values along some of the gene lineages are remarkable. For example, there have been 17 non-synonymous and 0 synonymous mutations that have accumulated during the divergence of the B5 gene (6 non-synonymous in the first half and 11 in the second half). The identical genes C1 and C2 have accumulated 25 non-synonymous changes and 0 synonymous changes since they shared a last common ancestor with the other genes of the ‘C’ clade, a stunningly intense episode of positive selection. Collapsing of the poorly supported node in each tree only marginally affects the results (Figure S4). Such extreme evolutionary patterns are unusual, but have been documented previously in other viral restriction factors due to the intense evolutionary arms races in which these genes are engaged [26], [28], [33], [34], [50].

These analyses can identify specific codon positions, and corresponding amino acid residues, that have repeatedly been subject to positive natural selection. Ten rapidly evolving codons were identified (Table S3), as illustrated with tick marks on the protein schematic in Figure 5A. Five of these fall in or near the RING domain (residues F48, V50, E54, E60, H69 in TRIM49/F1 coordinates), three in the coiled-coil domain (R166, C167, R222), and two in the B30.2 domain (Y320, A323).

Using secondary structure prediction and alignments to other TRIM proteins, we determined that rapidly evolving residues Y320 and A323 fall in a small loop (11–16 aa long) that lies between the second and third β-strands of the B30.2 domain (Figure S3). This surface-exposed “variable loop 1” (Figure 5B) has been rather well characterized, at least in the case of TRIM5α. In this protein, this loop is known to be a major determinant of recognition for retroviral capsids, and presumably constitutes the major binding interface with retroviruses. Sequence variation in this loop of TRIM5α accounts largely for the species-specific viral restriction patterns observed in various primate and mammalian species [28], [51]–[53]. It is hypothesized that the *TRIM5* gene has been engaged in an arms race with retroviruses throughout the diversification of mammals, and that natural selection has driven rapid sequence evolution of this loop for improved recognition of constantly changing retroviruses [47], [54]. Accordingly, there are multiple sites of positive selection in the variable loop 1 region of TRIM5α from primates [28], cows [27], and rabbits and hares [55], all of which restrict mammalian retroviruses. It is intriguing that the young *TRIM* genes identified here should have two codons evolving under positive selection in the B30.2 domain, with both of them falling in this small surface loop known to interact with retroviral capsids.

Three rapidly evolving residues were also identified in the coiled-coil domain (Figure 5A). In TRIM5α, the coiled-coil domain is the second domain that participates in retroviral target specificity [51], [56]. The rapidly evolving residues identified here are in regions shown to be critical in defining virus-specificity in TRIM5α [56], and known to contain codons evolving under positive selection in the *TRIM5* gene (Figure S3). In summary, positive selection has acted on these young *TRIM* genes in regions analogous to the major determinants of retroviral specificity in TRIM5α, suggesting that the novel genes could be retroviral restriction factors.

Based on the evolutionary signatures observed, we next tested whether these genes might encode retroviral restriction factors. We chose some of the genes with the highest branch-specific dN/dS values for functional testing, ones which could also be amplified in their complete form from human mRNA samples. These candidates (*A1*, *B1*, *B5*, *F1/TRIM49*, *F2*, and *F3*) are indicated with stars in Figure 5C. Except for *F1/TRIM49*, none of these genes have ever been previously studied. We tested the ability of these genes to restrict cellular entry of three different mammalian retroviruses which are known to be restricted by human and/or rhesus macaque TRIM5α: feline immunodeficiency virus (FIV), HIV, and murine leukemia virus (MLV). Interestingly, the young human *TRIM* genes did not restrict these retroviruses (Figure S6). If these genes do have anti-retroviral activity, these data suggest that their specificity is different than that of TRIM5α, consistent with the high degree of divergence observed in the regions that control target specificity. Perhaps these *TRIM* genes were honed to target retroviruses that are now extinct [57], [58]. Alternately, these *TRIM* genes may target other virus families altogether. For instance, *TRIM22* has experienced positive selection in these same retroviral targeting motifs, and while this gene may be relevant to retroviral infection [59], it also has activity against hepatitis B [60] and picornaviruses [61].

## Discussion

Here we identify and characterize what are likely to be the youngest *TRIM* genes in the human genome. While the ∼100 human *TRIM* genes are for the most part ancient, we now show that a substantial number of them (approximately 20%, not counting additional *TRIM* gene copies that are polymorphic in the human population) have arisen in recent evolutionary time, during the speciation of the great apes. Many of these genes have full-length open reading frames and produce spliced transcripts. Almost all have arisen from segmental duplications that can be traced to a single locus on the arm of chromosome 11. We propose that the segment 1 region on the arm of chromosome 11 is a *TRIM* gene “factory,” producing copies of the genes that it contains by spawning segmental duplications around the genome. Increased dosage of these genes may, in itself, be adaptive. Further, depending on the chromosomal context of new segmental duplications, the genes that they contain may be expressed in different tissues or at different developmental stages. However, we also find that positive selection is rapidly shaping the sequence of these genes, such that new copies may quickly become specialized for new functions or specificities. It will be important to determine what role, if any, these young *TRIM* genes play in innate immunity, particularly because these genes are copy number variable in human and primate populations.

Several insights into the evolutionary dynamics of large gene families can be gained from this study. *TRIM* genes are found throughout the human genome and the segmental duplications described here may illustrate one mechanism by which this family has expanded over time. Because all gene duplicates start out as polymorphisms segregating in populations, the observed copy number variation suggests that this gene family is still growing. Several lines of evidence support the idea that the fixation of new *TRIM* paralogs in the human genome has, at least in part, been an adaptive rather than a neutral process. First, *TRIM5* [28], *TRIM22* [27], *Pyrin/TRIM20* [46], and many of the *TRIM* genes described herein, have all evolved under positive selection, accumulating unexpectedly high numbers of non-synonymous mutations. New genes are especially prone to positive selection, probably because redundant gene copies provide templates for the evolution of new functions [24], [62]. Second, some *TRIM* genes have been highly dynamic in terms of species-specific gene gain and loss during mammalian evolution. For instance, cows and rodents possess independent, species-specific expansions of tandemly situated *TRIM5* paralogs, while dogs and cats have independently lost the function of this gene [27], [54], [63]. Likewise, the *TRIM* genes in segment 1 on chromosome 11 are also highly dynamic, having spawned at least two segmental duplications in African apes and another that is now polymorphic in the human population. Immunity genes are, in general, overrepresented amongst mammalian gene families that show rapid gene gain and loss, suggesting that these events are often adaptive [64]. Third, the acquisition of the young *TRIM* genes has not come without a cost to the human genome. Unequal crossing-over and aberrant homologous recombination between the tandem segments that contain *TRIM50*, *TRIM73*, and *TRIM74* causes Williams-Beuren syndrome in 1/7,500 to 1/25,000 newborns [65]. This fitness consequence might be expected to be offset by a fitness advantage, otherwise these regions would be selectively lost from the genome.

Historically, studies of human genetic variation have focused almost exclusively on single nucleotide polymorphism (SNP) differences between individuals. Recently, it has become apparent that large DNA segments are also commonly polymorphic between individuals, resulting from recent segmental duplications and deletions. CNV regions can be associated with disease, usually related to the altered gene dosage that they convey [66]. Another negative consequence of CNV duplications is that blocks of nearly identical sequence interspersed in a genome can create a volatile landscape for recombination. However, positive attributes can also be imagined for CNV regions, such as benefits that might be gained from increased dosage of certain genes. Such a fitness advantage has been suggested for the salivary amylase gene (*AMY1*), which is found in higher copy number in populations with higher starch diets [67]. Here we propose that CNV regions can also be a positive, adaptive force in genomes by driving the generation and diversification of gene families important to human immunity. For all of these reasons, studies of CNV regions are important for understanding individual disease susceptibility.

## Materials and Methods

### Gene sequences and phylogenetic analysis

Refseq annotated human coding sequences of genes of interest were downloaded from Genbank. Analysis of human – chimpanzee – orangutan – rhesus macaque orthogroups was performed by reciprocal-best hit analysis performed in the UCSC genome database [68]. Briefly, each human gene was used as a BLAT query [69] against the genome projects of the other species investigated. The top hits from these queries were then used to reciprocally query the human genome. All related sequences were then compiled and subjected to phylogenetic analysis. cDNA or full-gene sequences were aligned using MUSCLE as implemented in MEGA5 [70]. Alternate trees (neighbor joining, maximum likelihood, and maximum parsimony) were constructed within MEGA, with gapped positions excluded. Tree nodes were critically evaluated by performing 1,000 bootstrap replicates. The physical locations of the *TRIM* genes in the human (hg19), chimpanzee (panTro2), orangutan (ponAbe2), and rhesus macaque (rheMac2) genome assemblies were determined by manual inspection in the UCSC genome browser [68]. It was not possible to determine the structure of the chromosomal 11 loci in the marmoset genome assembly (calJac3), due to poor sequence quality.

### Gene expression analysis

Primers were designed to recognize novel *TRIM* genes (primer sequences are given in Table S7). Each primer set was designed to span at least one intron so that products resulting from processed transcripts could be differentiated from those potentially resulting from contaminating genomic DNA. SuperScript first-strand synthesis system for RT-PCR (Invitrogen) was used to synthesize cDNA from human testis total RNA (Clontech, 636533). PCR Supermix (Invitrogen) or *Ex Taq* polymerase (Takara) was used to amplify from cDNA. Individual PCR amplicons were cloned into vectors using the TOPO-TA Cloning kit (Invitrogen). For each sample, at least ten different colonies were randomly selected and were sequenced. These sequences have been submitted as records to Genbank (JF968445-JF968463).

### Characterizing patterns of molecular evolution

A sequence alignment was created from *TRIM* genes that have a full-length open reading frame (RING through B30.2). The tree length of this dataset is approximately 5 [73]. First, this alignment was checked for the signatures of gene conversion. If gene conversion of one paralog by another has occurred along the entire length of the two genes, this will not present a problem because a gene phylogeny will correctly reflect the fact that these two genes now have a very recent common ancestor and have been diverging only since the gene conversion event (although the record of previous evolutionary adaptations will have been erased in the converted gene). Problems occur when a gene conversion event has affected only part of a gene, as each gene half will then have a different location on the phylogenetic tree and no single tree will accurately represent the evolutionary history of the entire gene. To detect such events, the alignment was checked for phylogenetic incongruencies with the GARD program [49] implemented in Datamonkey [71]. Once the breakpoint had been identified, the tree structure for each half of the alignment was checked with multiple bootstrapping algorithms using MEGA5 as described above. Using the two halves of the alignment and the corresponding trees, maximum likelihood analysis was performed with codeml in the PAML 3.14.1 software package [72]. The multiple alignments were fitted to the NSsites models M1a, M7, M8a (null models) and M2a, M8 (positive selection models). Simulations were run with alternate models of codon frequencies (f3x4 and f61), and with multiple seed values for dN/dS (ω). Likelihood ratio tests were performed to assess whether positive selection models provide a significantly better fit to the data than null models. In situations where the null model could be rejected (p<0.05), posterior probabilities were assigned to individual codons belonging to the class of codons with dN/dS>1 with the Naive Empirical Bayes (NEB) algorithm implemented in codeml. The free ratio model (model 1, one dN/dS per branch) was also run in codeml to assess branch-specific values of dN/dS.

### Generation of stable cell lines

HA-tagged versions of human and rhesus *TRIM5* in the LPCX retroviral vector were obtained from the National Institutes of Health AIDS Research and Reference Reagent Program. *TRIM A1*, *B1*, *F1*, *F2*, and *F3* open reading frames were amplified from human cDNA using primers shown in Table S7. We were unable to amplify *B5* in its full length form, so in this case we fused the B30.2 domain of this gene to the tripartite domains of rhesus *TRIM5*. HA tags were fused to the C-terminus of each gene using PCR and these products were cloned into the LPCX retroviral vector (Clontech). Retroviruses containing these vectors were packaged in 293T cells by co-transfecting them along with the NB-MLV packing plasmid pCS2-mGP [74] and pC-VSV-G (provided by Hyeryun Choe). Supernatants were collected and used to infect CRFK cells purchased from American Type Culture Collection (ATCC) and grown in DMEM supplemented with 10% FBS. After 24 hours, media containing 8 µg/ml puromycin was added to select for transduced cells. Expression of TRIM proteins was detected by Western blot of 30 µg total protein using an anti-HA antibody (3F10, Roche, catalog 1867431).

### Viral entry assays

Viruses for single-cycle infection assays were packaged in 293T cells by co-transfection of plasmids encoding viral proteins and VSV-G, along with a transfer vector, as follows: N-MLV (pCIG3-N [75], pC-VSV-G, pLXCG:GFP), HIV-1 (pMDLg/pRRE, pRSV-Rev, pMD2.G, pRRLSIN.cPPT.PGK-GFP.WPRE; all available on Addgene), FIV (pFP93 [76], pC-VSV-G, pGINSIN:GFP [77]). After 48 hours, supernatant containing viruses was harvested, filtered, and frozen. For infection assays, CRFK stable cell lines were plated at a concentration of 5×10^4^ cells/well in a 12-well plate and infected with N-MLV, FIV, or HIV-1 the following day. Two days post-infection, cells were analyzed by flow cytometry for expression of GFP.

### Multiplex ligation-dependant probe amplification (MLPA) assay

We utilized the SALSA MLPA kit P200 Human DNA reference-1 and associated protocol (MRC-Holland, Amsterdam, The Netherlands). This kit includes the human control probes utilized. Our custom probe set was designed to contain eighteen pairs of MLPA probes spanning segment 1 (Table S4). These probes also perfectly match paralogous regions in segment 3, due to the fact that these segments are nearly identical, but are designed to contain at least two mismatches to all other paralogous sequences located on chromosomes 2 or 11 (or anywhere else in the human genome, as determined by BLAT searching on the UCSC human genome browser). Probes were positioned both in genes (8 probe pairs) and in intergenic regions (10 probe pairs). The average distance between probe pairs is 9304 bp. PCR primers supplied with this kit were fluorescently labeled with FAM, and FAM-labeled fragments obtained from each experiment were analyzed with an Applied Biosystems 3730 DNA analyzer. Peak spectra were checked for quality in two ways. First, the spectra were analyzed with the ABI software Peak Scanner (v.1.0) to evaluate the fragment size quality using a size standard that was included during fragment analysis (500ROX, ABI). If the quality flag indicated “pass,” the samples including their fragment size information were exported as a combined table. Second, the MRC-Holland software Coffalyzer (v.8) was used to evaluate the signals of the control probes supplied with the MLPA kit. Controls are designed to confirm sufficient amounts of template DNA and completion of DNA denaturation and ligation steps. Finally, GeneMarker (v.1.7) software was used to normalize and analyze MLPA experiments that passed both of these quality control steps. Advanced population normalization was used and MLPA analysis settings were as follows: MLPA ratio (analysis method), adjustment by control probes, and quantification by peak height. After normalization of fragment data to the reference genome (sample NA10851 from Utah), duplications and deletions were defined as probes that gave a signal intensity of >1.35 (duplication) or <0.65 (deletion) that of the reference genome. Because the samples analyzed were a mixture of male and female samples, control probes on the X and Y chromosomes were used to show that these enrichment and depletion thresholds are robust in predicting gain and loss of control targets located on sex chromosomes (Table S5). Because false signals may be caused by unknown SNPs at the target locus or elsewhere, signals of enrichment or depletion seen only with a single probe pair were disregarded.

## Supporting Information

Figure S1 Sequence alignments of the TRIM RING and B-box 2 domains. Of the 31 human *TRIM* genes identified on chromosomes 11 and 2, eleven appear to have been pseudogenized based on the acquisition of frame shifts or stop codons. The predicted RING and B-box2 domains encoded by the other 20 genes are diagrammed here. Bold characters highlight consensus motifs of the RING and B-box 2 domains [21]. All but one of the predicted RING domains, that in G3, has retained the signature (C-x_2_-C-x_11–16_-C-x-H-x_2_-C-x_2_-C-x_7–74_-C-x_2_-[C/D]) motif characteristic of the TRIM family RING domain. The B-box 2 domain, defined by the signature (C-x_2_-H-x_7–9_-C-x_2_-[C/D/H/E]-x_4_-C-x_2_-C-x_3–6_-H-x_2–4_-[C/H]) motif, is conserved in all but the C1 and C2 genes. Asterisks along the bottom indicate positions of strict sequence conservation.
(PDF)

Figure S2 Domain structures and expression evidence for novel *TRIM* genes on chromosomes 2 and 11. (A) Of the 31 human *TRIM* genes identified on chromosomes 11 and 2, eleven appear to have been pseudogenized based on the acquisition of frame shifts or stop codons. Predicted cDNA sequences for the remaining 20 were translated into amino acid sequence, and the signature protein domains of the TRIM family were identified and illustrated to scale with domain diagrams. The TRIM RING and B-box domains are defined in Figure S1. Coiled-coil and B30.2 domains were identified with the secondary structure prediction program on the JPRED server (http://www.compbio.dundee.ac.uk/www-jpred/) [81]. The coiled-coil is easily identified as one long alpha helix, while the B30.2 domain is comprised of a string of 13 tandem beta strands [82]. Asterisks in the G3, C1, C2 diagrams indicate deviations from the strict consensus sequences of the RING and B-box 2 domains (see Figure S1). Next to each schematic, a star indicates that processed mRNA transcripts for that gene have been reported, either in Genbank (black stars) or in our studies shown in panel B (white stars). (B) Primers were designed to amplify *TRIM* transcripts from cDNA prepared from human testes. Universal primers were designed for each of the phylogenetic subclades of *TRIM* genes discussed in the paper. Primer sequences are reported in Table S7. Primers were designed to span introns so that both expression and splicing could be verified, but only small portions (245–1368 bp) were amplified, resulting in fragment length differences seen in the gel. Because of the sequence similarity among the genes in each subclade, most PCR reactions amplified more than one *TRIM* paralog. For this reason, at least ten different fragments from each PCR pool were cloned, sequenced, and examined for diagnostic mutations that unambiguously distinguish each of these genes from the others. The genes found to be amplified in each PCR pool are listed above the gel, with expressed pseudogenes indicated in parenthesis. In all, processed transcripts were detected for 11 of the non-pseudogenized *TRIM* genes (indicated with open stars in panel A).
(PDF)

Figure S3 TRIM residues under positive selection. (A) An alignment of the first part of the B30.2 domain is shown. One sequence is shown from each subclade of the novel *TRIM* genes included in the evolutionary analysis. The positions of the two residues under positive selection are highlighted in yellow. Because the B30.2 is a motif where structure is more highly conserved than sequence [83], the alignment was aided by predicted secondary structural elements. Predicted beta strands are underlined and the consensus locations of the beta strands (green boxes) are in agreement with crystal structures of the B30.2 domain [82], [83]. The “variable loop 1” region between beta strands 2 and 3 is notoriously poorly conserved in both sequence and length [28], [82], [84] and the exact alignment of residues in this region is not possible to determine. Residues under positive selection have previously been found in the variable loop 1 of TRIM5α and TRIM22 [27], [28], and these are indicated in yellow. (B) An alignment is shown of the entire coiled-coil domain, with amino acid positions identified as evolving under positive selection highlighted in yellow. Residues previously identified as subject to positive selection in TRIM5α and TRIM22 are also highlighted in yellow [27], [28]. Pink balls indicate residues previously found to contribute to retroviral target specificity in TRIM5α [56]. Boxes show regions of the coiled-coil that may be critical to retroviral targeting, based on evolutionary and genetic signatures summarized here. In both panels, asterisks mark perfectly conserved residues.
(PDF)

Figure S4 Phylogenetic trees used in PAML analyses, along with a full summary of free ratio results. All trees used in the evolutionary analyses are shown. The sequence alignment was divided at a phylogenetic breakpoint that was detected between the RING and B-box 2 domains. The trees made from each half of the alignment (tree 3 and tree 1) are shown. In each case, there was one weakly supported node (green branch) that was collapsed to yield tree 4 and tree 2. All evolutionary analyses were verified using both trees, and the results of the PAML free ratio analysis are shown here for all trees. The dN/dS value is shown along each branch, along with the predicted number of non-synonymous and synonymous changes (N∶S) that occurred along each branch. Text is in red where dN/dS >1 or, arbitrarily, where N∶S≥3∶0 in cases where dS = 0. The NSsites models were also verified with all possible trees, as shown in Table S3.
(PDF)

Figure S5 Summary of previously published CNVs in the region of segments 1/3 (11q14.3). A schematic of the region of segment 1/segment 3 is shown along the top, with information from three structural variation data tracks from the UCSC genome database aligned directly beneath. Information from the latter two tracks has been re-drawn for readability. The “RefSeq Genes” track shows that some of these genes have been previously annotated, although in some cases there is redundancy because the genes in each segment are so similar in sequence. The “Segmental Dups” track shows that the Eichler Lab has previously detected this tandem duplication event. The “Database of Genomic Variants (DGV) Track” shows major CNV events reported in this region. A key to each numbered CNV event (ie “3862”) is shown in the table at the bottom.
(PDF)

Figure S6 Novel TRIM proteins do not inhibit entry of three viruses targeted by TRIM5α. (A) Western blot of whole cell lysates from CRFK stable cell lines expressing the *TRIM* genes selected for functional analyses. As a negative control, CRFK cell lines were transduced with the empty LPCX vector. Stable cell lines expressing human (Hs) and rhesus (Rh) TRIM5α were included as controls. (B-D) Infection of stable cell lines with (B) HIV-1, (C) FIV, or (D) N-MLV was assessed by GFP fluorescence using flow cytometry, as each virus carries a GFP reporter gene. In all panels, the asterisk (*) indicates that the B30.2 domain of *B5* was fused to the tripartite domains of rhesus *TRIM5*.
(PDF)

Table S1 Percent identity between segmental duplications on chromosome 11. Each duplicated region was aligned using BioEdit. The alignment was imported into DNASTAR's MegAlign program in the Lasergene version 5.0 software package (http://www.dnastar.com) and sequence identity was calculated.
(PDF)

Table S2 Evolutionary divergence between open reading frames (ORFs) for the 20 intact human genes identified on chromosomes 2 and 11. The fraction of base differences between each set of sequences is shown. All positions containing gaps were eliminated. Distances were calculated with MEGA5 [70].
(PDF)

Table S3 Summary of codeml simulations conducted with PAML.
(PDF)

Table S4 MLPA probe sequences. LPO and RPO stand for left probe oligo and right probe oligo, respectively. Green and blue letters indicate forward and reverse universal primer binding sites, respectively. Lower case letters represent stuffer sequences that are built into each probe pair so that it produces a PCR product of unique size.
(PDF)

Table S5 Results of MLPA analysis for all 72 genomes surveyed. MLPA ratios (normalized to Utah 10851) are shown for each probe pair for each individual. Deletions and duplications are indicated where ratios are <0.65 (red highlight) and >1.35 (green highlight), respectively. These cut-off ratios are commonly used (*see* MLPA validation report (www.eurogentest.org)). Enrichments and depletions specific to a single probe have been ignored.
(XLSX)

Table S6 Human individuals surveyed in the MLPA analysis. All genomic DNA samples were obtained from Coriell except where indicated.
(PDF)

Table S7 Primers used in this study. Primers are shown for the amplification of *TRIM* transcripts from cDNA. Also shown are primers used to amplify full-length genes for viral restriction assays.
(PDF)
