# Supplementary material for: Identification of a Genomic Reservoir for New TRIM Genes in Primate Genomes
Source: PLoS Genet. 2011 Dec 1;7(12):e1002388. doi: 10.1371/journal.pgen.1002388 (PMC3228819; doi:10.1371/journal.pgen.1002388)
Supplement: Figure S1 — Sequence alignments of the TRIM RING and B-box 2 domains. Of the 31 human TRIM genes identified on chromosomes 11 and 2, eleven appear to have been pseudogenized based on the acquisition of frame shifts or stop codons. The predicted RING and B-box2 domains encoded by the other 20 genes are diagrammed here. Bold characters highlight consensus motifs of the RING and B-box 2 domains [21]. All but one of the predicted RING domains, that in G3, has retained the signature (C-x2-C-x11–16-C-x-H-x2-C-x2-C-x7–74-C-x2-[C/D]) motif characteristic of the TRIM family RING domain. The B-box 2 domain, defined by the signature (C-x2-H-x7–9-C-x2-[C/D/H/E]-x4-C-x2-C-x3–6-H-x2–4-[C/H]) motif, is conserved in all but the C1 and C2 genes. Asterisks along the bottom indicate positions of strict sequence conservation. (PDF) [file pgen.1002388.s001.pdf]

Han et al, Figure S1

```
                             Ring Domain
A1          MNSGISQVFQRELTCPICLNYFIDPVTIDCGHSFCRPCFYLNWQDIPILTQCFECLKTTQQRNLKTNI(68)
A2          MNSGISQVFQRELTCPICLNYFIDPVTIDCGHSFCRPCFYLNWQDIPILTQCFECLKTTQQRNLKTNI
A3(TRIM48)  MNSGISQVFQRELTCPICMNYFIDPVTIDCGHSFCRPCFYLNWQDIPILTQCFECIKTIQQRNLKTNI
B1          MDSDDLQVFQNELICCICVNYFIDPVTIDCGHSFCRPCLCLCSEEGRAPMRCPSCRKTSEKPNFNTNL
B2(TRIM64)  MDSDDLQVFQNELICCICVNYFIDPVTIDCGHSFCRPCLCLCSEEGRAPMRCPSCRKISEKPNFNTNV
B5          MDSDDLQVFQNELICCICVNYFIDPVTIDCGHSFCRPCLCLCSEEGRAPMRCPSCRKISEKPNFNTNV
C1          MNSGILQVFQRALTCPICMNYFIDPVTIDCGHNFCRPCFYLNWRDMAVLAQCSKCKKTIQQRNLKTDI
C2          MNSGILQVFQRALTCPICMNYFIDPVTIDCGHNFCRPCFYLNWRDMAVLAQCSKCKKTIQQRNLKTDI
C6(TRIM51)  MNSGILQVFQRALTCPICMNYFLDPVTIDCGHSFCRPCLYLNWQDTAVLAQCSECKKTTRQRNLNTDI
C8          MNSGILQVFQRELICPICMNYFIDPVTIDCGHSFCRPCFYLNWQDMAVLAQCSKCKKTTRQRNLKTNI
D1(TRIM53)  MNSGISQDFQMEITCPICMNYFIDPVTIDCGHSFCRPCFYFNWQDIPILTQCFECMKTTWQRNLKTNI
D2          MNSGISQDFQMEITCPICMNYFIDPVTIDCGHSFCRPCFYFNWQDIPILTQCFECMKTTWQRNLKTNI
D3          MNSGILQEFQMELTCPICMKYFIDPVTVDCGHSFCRPCFYFKWQDIPIFTQCFECMKTTWQRNLKTNI
F1(TRIM49)  MNSGILQVFQGELICPLCMNYFIDPVTIDCGHSFCRPCFYLNWQDIPFLVQCSECTKSTEQINLKTNI
F2          MNSGILQVFQGELICPLCMNYFIDPVTIDCGHSFCRPCFYLNWQDIPFLVQCSECTKSTEQINLKTNI
F3          MNSGILQVFQRELICPICMNYFIDPVTIDCGHSFCRPCFYLNWKDSPFLVQCSECTKSTGQINLKTNI
G1(TRIM77)  MASAITQCSTSELTCSICTDYLTDPVTICCGHRFCSPCLCLLWEDTLTPNCCPVCREISQQMYFKRII
G3          MASAITQCSTSELTCSICTVYLTDPVTIGCGHRFCSPCLCLLWEDPLTPNCCPGRREISQQMYFKRIT
H1          MDSDFSHAFQKELTCVICLNYLVDPVTICCGHSFCRPCLCLSWEEAQSPANCPACREPSPKMDFKTNI
H2(TRIM43)  MDSDFSHAFQKELTCVICLNYLVDPVTICCGHSFCRPCLCLSWEEAQSPANCPACREPSPKMDFKTNI
            * *           *  *  *  ****  * * ** **             *

                           B-box 2 Domain
A1          RLKKMASRARKASLWLFLSSEEQMCGTHRETKKIFCEVDRSLLCLLCSSSLEHRYHRHCPAEWAAEEH(136)
A2          RLKKMASRARKASLWLFLSSEEQMCGTHRETKKIFCEVDRSLLCLLCSSSLEHRYHRHCPAEWAAEEH
A3(TRIM48)  RLKKMASLARKASLWLFLSSEEQMCGIHRETKKMFCEVDRSLLCLLCSSSQEHRYHRHCPAEWAAEEH
B1          VLKKLSSLARQTRPQNINSSD-NICVLHEETKELFCEADKRLLCGPCSESPEHMAHSHSPIGWAAEEC
B2(TRIM64)  VLKKLSSLARQTRPQNINSSD-NICVLHEETKELFCEADKRLLCGPCSESPEHMAHSHSPIGWAAEEC
B5          ALKKLASLARQTRPQNINSSD-NICVLHEETKELFCEADKRLLCGPCSESPEHMAHSHSPIGWAAEEC
C1          CLKNMASTARKASLWQFLSSEEQICGMHRETKKMFCEVNKSRLCWLCSNSQEHRNHRHCPIEWAAEER
C2          CLKNMASTARKASLWQFLSSEEQICGMHRETKKMFCEVNKSRLCWLCSNSQEHRNHRHCPIEWAAEER
C6(TRIM51)  CLKNMAFIARKASLRQFLSSEEQICGMHRETKKMFCEVDKSLLCLPCSNSQEHRNHIHCPIEWAAEER
C8          CLKNMASIARKASLRQFLSSEEQICGTHRETKEMFCEVDKSLLCLLCSNSQEHRNHRHCPTEWAAEER
D1(TRIM53)  HLKQMASLARKASLWLFLSSEEQMCGTHRETKKIFCEVDRSLLCLLCSSSQEHRYHRHRPIEWAAEEH
D2          HLKQMASLARKASLWLFLSSEEQMCGTHRETKKIFCEVDRSLLCLLCSSSQEHRYHRHRPIEWAAEEH
D3          HLKKMASLARKVSLWLFLSSEEQMCGTHRETKKMFCEVDKSLLCLLCSSSQEHRYHRHRPVEWAAEEH
F1(TRIM49)  HLKKMASLARKVSLWLFLSSEEQMCGTHRETKKIFCEVDRSLLCLLCSSSQEHRYHRHRPIEWAAEEH
F2          HLKKMASLARKVSLWLFLSSEEQMCGTHRETKKIFCEVDRSLLCLLCSSSQEHRYHRHRPIEWAAEEH
F3          HFKKMASLARKVSLWLFLSSEEQMCGTHRETKKMFCEVDRSLLCLLCSSSQEHRDHRHCPIESAAEEH
G1(TRIM77)  FAEKQVIPTRESVPCQLSSSAMLICRRHQEIKNLICETDRSLLCFLCSQSPRHATHKHYMTREADEYY
G3          FAEKQVIPTRESVSCQLSSSAMLICRRHQEIKNLICETDRSLLCFLCSQSPRHATHKHYMTKEADEYY
H1          LLKNLVTIARKASLWQFLSSEKQICGTHRQTKKMFCDMDKSLLCLLCSNSQEHGAHKHYPIEEAAEED
H2(TRIM43)  LLKNLVTIARKASLWQFLSSEKQICGTHRQTKKMFCDMDKSLLCLLCSNSQEHGAHKHHPIEEAAEEH
                     *         *    *  *   *   *      **  ** *  *  * *     * *
```
