# Supplementary figures and images for: Identification of a Genomic Reservoir for New TRIM Genes in Primate Genomes
Source: PLoS Genet. 2011 Dec 1;7(12):e1002388. doi: 10.1371/journal.pgen.1002388 (PMC3228819; doi:10.1371/journal.pgen.1002388)

# Han et al, Figure S2

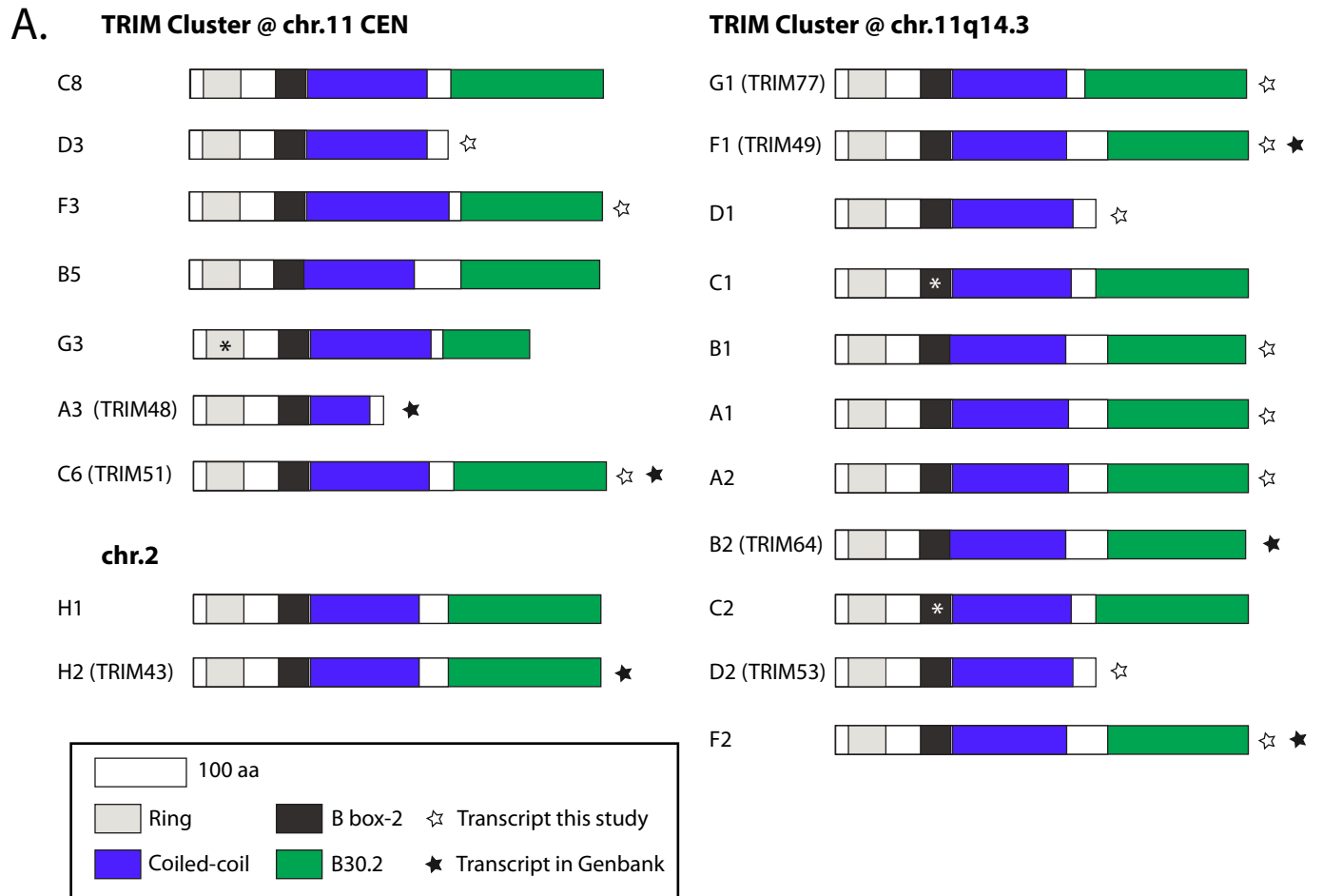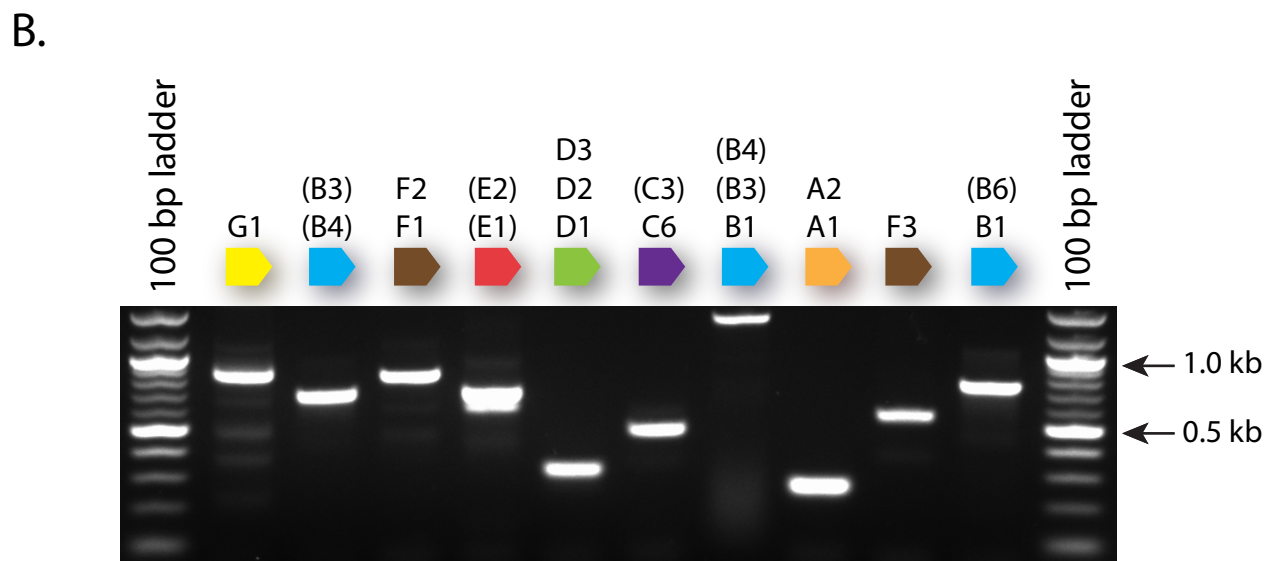

Supplement: Figure S2 — Domain structures and expression evidence for novel TRIM genes on chromosomes 2 and 11. (A) Of the 31 human TRIM genes identified on chromosomes 11 and 2, eleven appear to have been pseudogenized based on the acquisition of frame shifts or stop codons. Predicted cDNA sequences for the remaining 20 were translated into amino acid sequence, and the signature protein domains of the TRIM family were identified and illustrated to scale with domain diagrams. The TRIM RING and B-box domains are defined in Figure S1. Coiled-coil and B30.2 domains were identified with the secondary structure prediction program on the JPRED server (http://www.compbio.dundee.ac.uk/www-jpred/) [81]. The coiled-coil is easily identified as one long alpha helix, while the B30.2 domain is comprised of a string of 13 tandem beta strands [82]. Asterisks in the G3, C1, C2 diagrams indicate deviations from the strict consensus sequences of the RING and B-box 2 domains (see Figure S1). Next to each schematic, a star indicates that processed mRNA transcripts for that gene have been reported, either in Genbank (black stars) or in our studies shown in panel B (white stars). (B) Primers were designed to amplify TRIM transcripts from cDNA prepared from human testes. Universal primers were designed for each of the phylogenetic subclades of TRIM genes discussed in the paper. Primer sequences are reported in Table S7. Primers were designed to span introns so that both expression and splicing could be verified, but only small portions (245–1368 bp) were amplified, resulting in fragment length differences seen in the gel. Because of the sequence similarity among the genes in each subclade, most PCR reactions amplified more than one TRIM paralog. For this reason, at least ten different fragments from each PCR pool were cloned, sequenced, and examined for diagnostic mutations that unambiguously distinguish each of these genes from the others. The genes found to be amplified in each PCR pool are listed above the ge [file pgen.1002388.s002.pdf]

**A.**

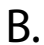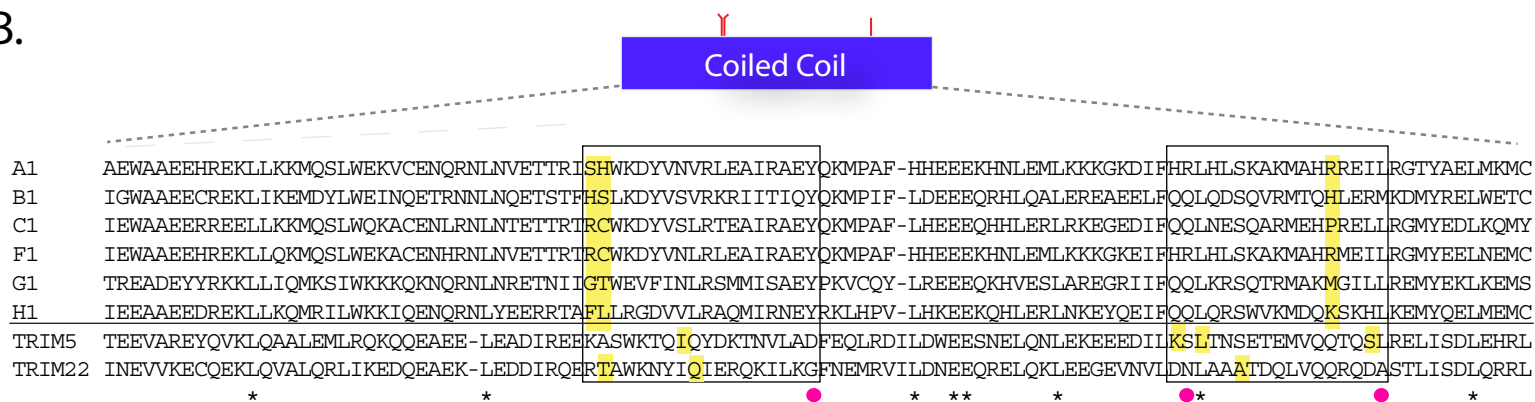

Supplement: Figure S3 — TRIM residues under positive selection. (A) An alignment of the first part of the B30.2 domain is shown. One sequence is shown from each subclade of the novel TRIM genes included in the evolutionary analysis. The positions of the two residues under positive selection are highlighted in yellow. Because the B30.2 is a motif where structure is more highly conserved than sequence [83], the alignment was aided by predicted secondary structural elements. Predicted beta strands are underlined and the consensus locations of the beta strands (green boxes) are in agreement with crystal structures of the B30.2 domain [82], [83]. The “variable loop 1” region between beta strands 2 and 3 is notoriously poorly conserved in both sequence and length [28], [82], [84] and the exact alignment of residues in this region is not possible to determine. Residues under positive selection have previously been found in the variable loop 1 of TRIM5α and TRIM22 [27], [28], and these are indicated in yellow. (B) An alignment is shown of the entire coiled-coil domain, with amino acid positions identified as evolving under positive selection highlighted in yellow. Residues previously identified as subject to positive selection in TRIM5α and TRIM22 are also highlighted in yellow [27], [28]. Pink balls indicate residues previously found to contribute to retroviral target specificity in TRIM5α [56]. Boxes show regions of the coiled-coil that may be critical to retroviral targeting, based on evolutionary and genetic signatures summarized here. In both panels, asterisks mark perfectly conserved residues. (PDF) [file pgen.1002388.s003.pdf]

# Han et al, Figure S4

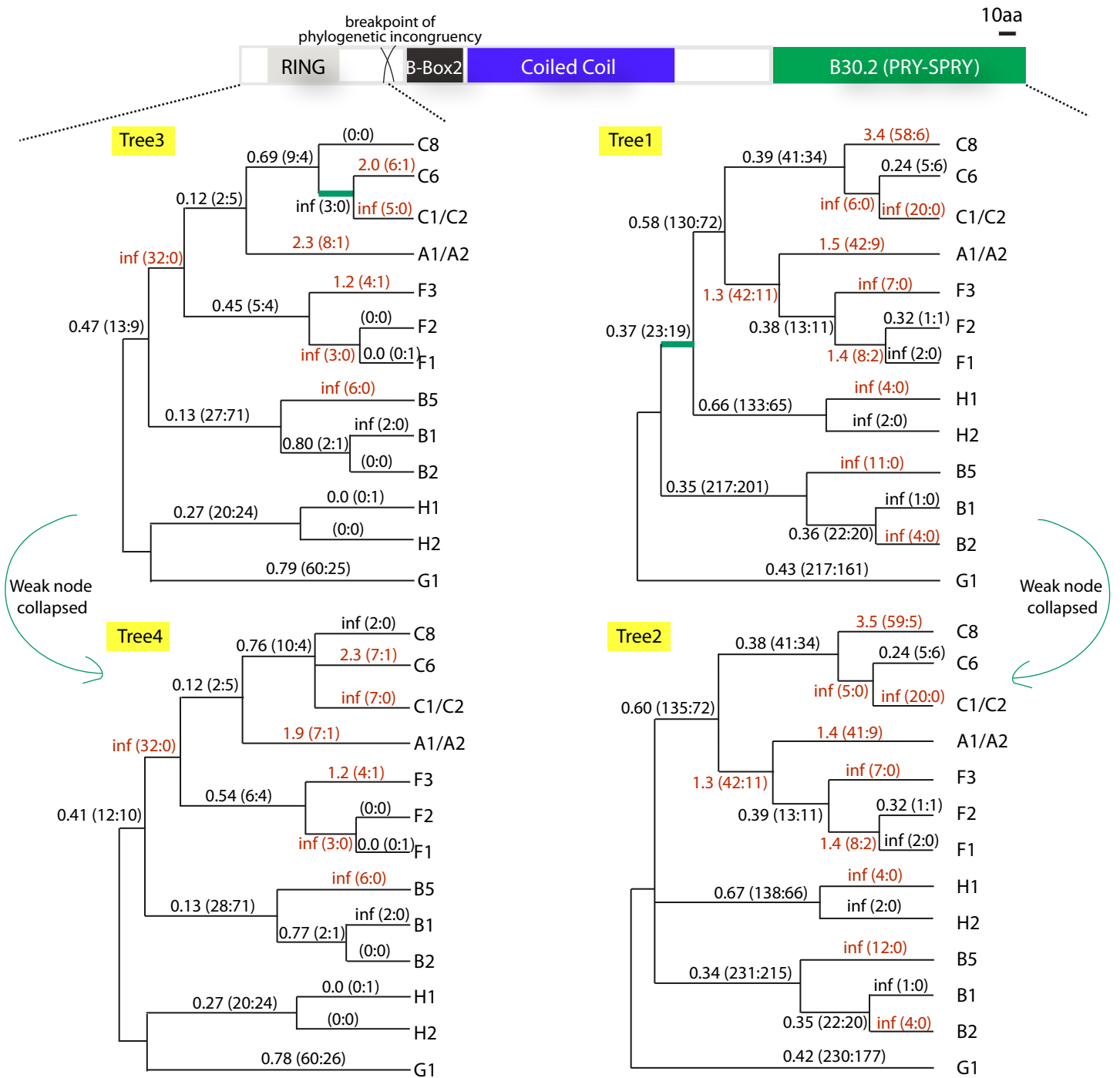

Supplement: Figure S4 — Phylogenetic trees used in PAML analyses, along with a full summary of free ratio results. All trees used in the evolutionary analyses are shown. The sequence alignment was divided at a phylogenetic breakpoint that was detected between the RING and B-box 2 domains. The trees made from each half of the alignment (tree 3 and tree 1) are shown. In each case, there was one weakly supported node (green branch) that was collapsed to yield tree 4 and tree 2. All evolutionary analyses were verified using both trees, and the results of the PAML free ratio analysis are shown here for all trees. The dN/dS value is shown along each branch, along with the predicted number of non-synonymous and synonymous changes (N∶S) that occurred along each branch. Text is in red where dN/dS >1 or, arbitrarily, where N∶S≥3∶0 in cases where dS = 0. The NSsites models were also verified with all possible trees, as shown in Table S3. (PDF) [file pgen.1002388.s004.pdf]

Han et. al.  
Figure S6

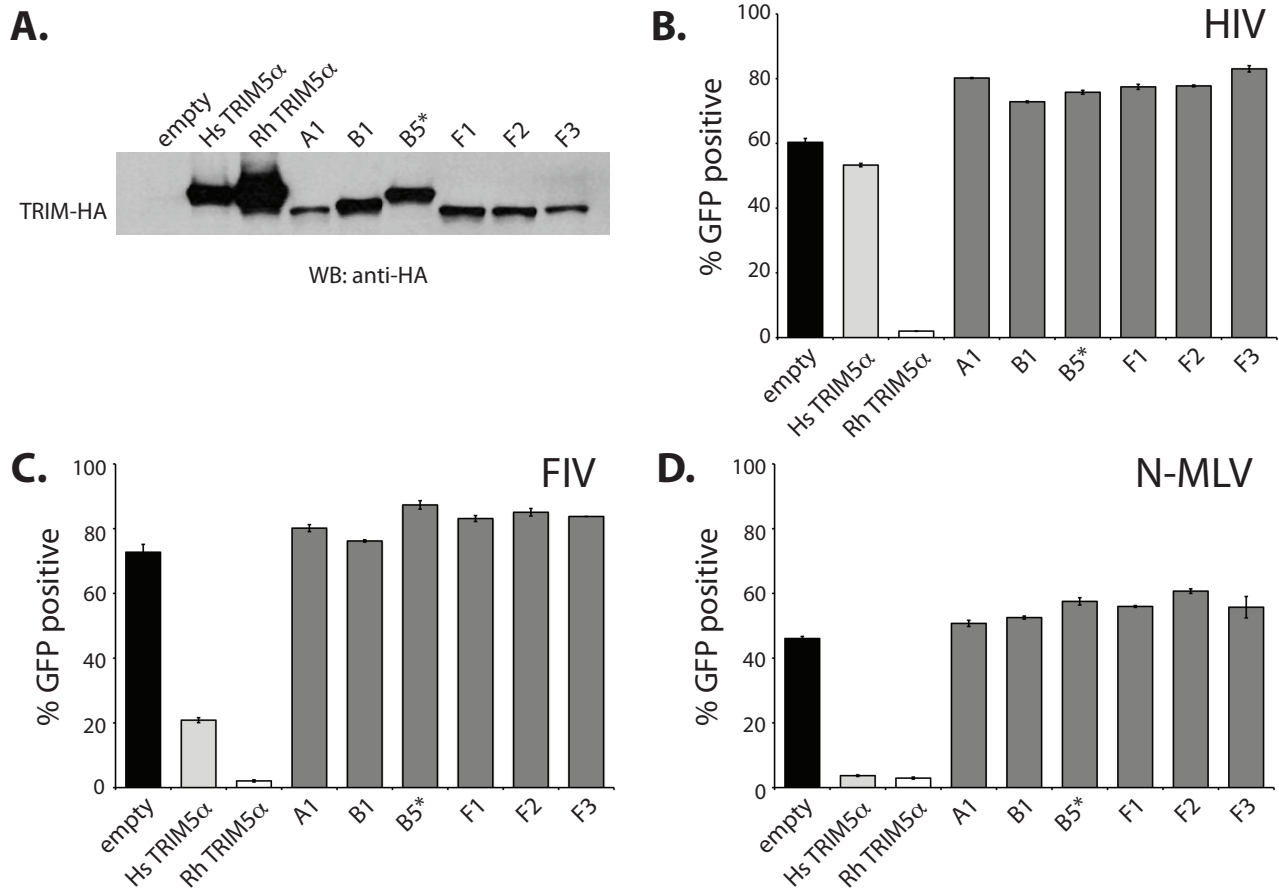

Supplement: Figure S6 — Novel TRIM proteins do not inhibit entry of three viruses targeted by TRIM5α. (A) Western blot of whole cell lysates from CRFK stable cell lines expressing the TRIM genes selected for functional analyses. As a negative control, CRFK cell lines were transduced with the empty LPCX vector. Stable cell lines expressing human (Hs) and rhesus (Rh) TRIM5α were included as controls. (B-D) Infection of stable cell lines with (B) HIV-1, (C) FIV, or (D) N-MLV was assessed by GFP fluorescence using flow cytometry, as each virus carries a GFP reporter gene. In all panels, the asterisk (*) indicates that the B30.2 domain of B5 was fused to the tripartite domains of rhesus TRIM5. (PDF) [file pgen.1002388.s006.pdf]
