# Supplementary material for: Identification of a Genomic Reservoir for New TRIM Genes in Primate Genomes
Source: PLoS Genet. 2011 Dec 1;7(12):e1002388. doi: 10.1371/journal.pgen.1002388 (PMC3228819; doi:10.1371/journal.pgen.1002388)
Supplement: Figure S5 — Summary of previously published CNVs in the region of segments 1/3 (11q14.3). A schematic of the region of segment 1/segment 3 is shown along the top, with information from three structural variation data tracks from the UCSC genome database aligned directly beneath. Information from the latter two tracks has been re-drawn for readability. The “RefSeq Genes” track shows that some of these genes have been previously annotated, although in some cases there is redundancy because the genes in each segment are so similar in sequence. The “Segmental Dups” track shows that the Eichler Lab has previously detected this tandem duplication event. The “Database of Genomic Variants (DGV) Track” shows major CNV events reported in this region. A key to each numbered CNV event (ie “3862”) is shown in the table at the bottom. (PDF) [file pgen.1002388.s005.pdf]

Han et al, Figure S5

Compilation of Structural Variation Data Tracks (UCSC) in the Segment 1/Segment 3 Region

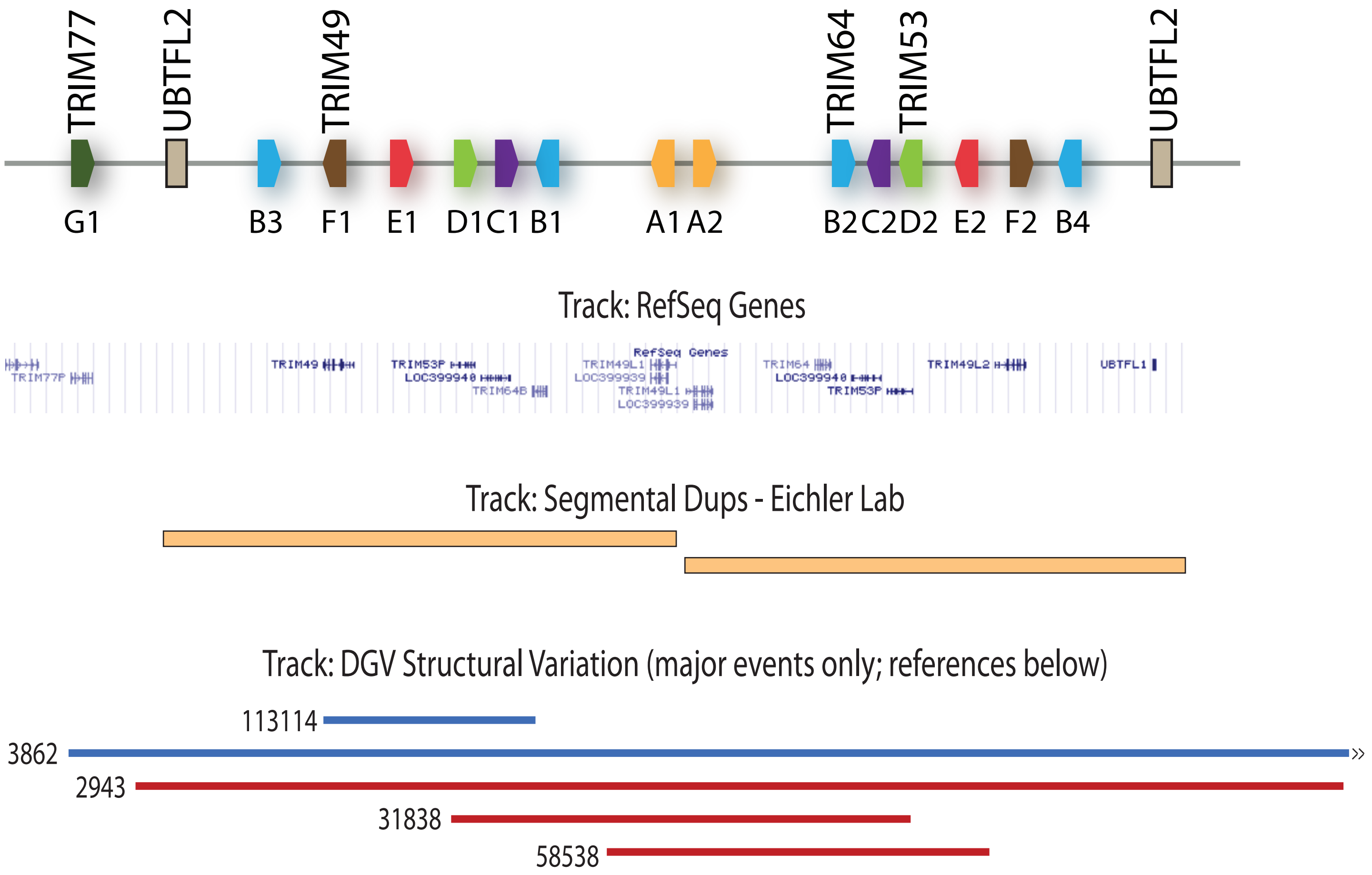

CNV code	Size (bp)	Gain/Loss	Sample	Paper
113114	73,170	Gain	NA18997, NA18969 (Japanese); AK6 (Korean)	Park et al (2010) Nature Genetics
3862	487,151	Gain	NA18573 (Chinese) - also detected this study	Redon et al (2006) Nature
2943	419,315	Loss	NA18622 (Chinese)	Redon et al (2006) Nature
31838	159,783	Loss	NA18502 (Yoruban)	Perry et al (2008) Am J Hum Gen
58538	132,269	Loss	AK1 (Korean)	Kim et al (2009) Nature
