# Supplementary material for: Identification of a Genomic Reservoir for New TRIM Genes in Primate Genomes
Source: PLoS Genet. 2011 Dec 1;7(12):e1002388. doi: 10.1371/journal.pgen.1002388 (PMC3228819; doi:10.1371/journal.pgen.1002388)
Supplement: Table S1 — Percent identity between segmental duplications on chromosome 11. Each duplicated region was aligned using BioEdit. The alignment was imported into DNASTAR's MegAlign program in the Lasergene version 5.0 software package (http://www.dnastar.com) and sequence identity was calculated. (PDF) [file pgen.1002388.s007.pdf]

Supplemental Table S1. Sequence identity between segmental duplictions

Locus	Identity (%)	Aligment length (bp)[a]
**Segment 1 vs. Segment 3**		
B3 vs. B4	99.7	5398
F1 vs. F2	99.4	6344
E1 vs. E2	99.7	6855
D1 vs. D2	99.9	4434
C1 vs. C2	99.9	7944
B1 vs. B2	99.5	5392
A1 vs. A2	100	6124
5' FR vs. 3' FR	99.2	34525
F1-B3 IR vs. F2-B4 IR	99.3	15761
E1-F1 IR vs. E2-F2 IR	99.5	10705
D1-E1 IR vs. D2-E2 IR	99.6	17851
C1-D1 IR vs. C2-D2 IR	99.9	5746
B1-C1 IR vs. B2-C2 IR	99.7	7727
A1-B1 IR vs. A2-B2 IR	99.7	35572
3' FR vs. 5' FR	100	2913
**Average**	**99.64**	**170378**[b]
**Segment 1 vs. Segment 2**		
G1 vs. G3	97	7563
B3 vs. B5	95.6	5407
F1 vs. F3	97.5	6355
E1 vs. E3	96.4	5726
D1 vs. D3	95.9	4427
C1 vs. C8	94.5	6207
5' FR vs. 3' FR[c]	97.3[c]	158718
B3-G1 IR vs. B5-G3 IR	96.2	52802
F1-B3 IR vs. F3-B5 IR	97.2	15748
E1-F1 IR vs. E3-F3 IR	97.1	15635
D1-E1 IR vs. D3-E3 IR	96.2	17578
C1-D1 IR vs. C8-D3 IR	94.8	5766
3' FR vs. 5' FR	95.3	555
**Average**	**96.2**	**302487**[b]

FR and IR stand for flanking region and intergenic region, respectively.
Gaps greater than 5bp are omitted.

[a] Aligment length includes gaps

[b] Total alignment length

[c] Identity of this long region was calculated with BLAT on the UCSC browser
