# Supplementary material for: Identification of a Genomic Reservoir for New TRIM Genes in Primate Genomes
Source: PLoS Genet. 2011 Dec 1;7(12):e1002388. doi: 10.1371/journal.pgen.1002388 (PMC3228819; doi:10.1371/journal.pgen.1002388)
Supplement: Table S2 — Evolutionary divergence between open reading frames (ORFs) for the 20 intact human genes identified on chromosomes 2 and 11. The fraction of base differences between each set of sequences is shown. All positions containing gaps were eliminated. Distances were calculated with MEGA5 [70]. (PDF) [file pgen.1002388.s008.pdf]

Supplemental Table S2: Genetic distances between pairs of sequences
(open reading frame only)

ORF 1	ORF2	Dist
C1	C2	0.000
A1	A2	0.000
D1	D2	0.000
F1	F2	0.002
B2	B1	0.005
H2	H1	0.007
F3	F2	0.018
F1	F3	0.020
A1	A3	0.021
A2	A3	0.021
G1	G3	0.027
B2	B5	0.035
D1	D3	0.035
D2	D3	0.035
B1	B5	0.037
A1	D1	0.039
A2	D1	0.039
A3	D1	0.039
A1	D2	0.039
A2	D2	0.039
A3	D2	0.039
C1	C6	0.048
C2	C6	0.048
A3	D3	0.053
A1	D3	0.053
A2	D3	0.053
F2	D1	0.055
F2	D2	0.055
F2	A1	0.057
F2	A2	0.057
F1	D1	0.057
F1	D2	0.057
F1	A1	0.059
F1	A2	0.059
F2	A3	0.060
C6	C8	0.062
F1	A3	0.062
C1	C8	0.062
C2	C8	0.062
F3	D1	0.069
F3	D2	0.069
F3	A1	0.071
F3	A2	0.071
F3	A3	0.071
F2	D3	0.073
F1	D3	0.074
F3	D3	0.083
C8	A1	0.124
C8	A2	0.124
C8	A3	0.126

C8	D1	0.126
C8	D2	0.126
C8	F2	0.128
C1	A3	0.128
C2	A3	0.128
C8	F1	0.129
C1	F2	0.129
C2	F2	0.129
C1	F1	0.131
C2	F1	0.131
C6	F1	0.131
C6	F2	0.131
C1	A1	0.131
C2	A1	0.131
C1	A2	0.131
C2	A2	0.131
C6	D1	0.131
C6	D2	0.131
C8	F3	0.133
C6	A3	0.133
C1	D1	0.133
C2	D1	0.133
C1	D2	0.133
C2	D2	0.133
C6	F3	0.135
C6	A1	0.135
C6	A2	0.135
C8	D3	0.135
C1	F3	0.138
C2	F3	0.138
C6	D3	0.138
C1	D3	0.140
C2	D3	0.140
H2	D1	0.236
H2	D2	0.236
H2	F2	0.236
H2	F1	0.238
H2	F3	0.239
H1	F3	0.239
H1	F2	0.239
H1	D1	0.239
H1	D2	0.239
H2	A1	0.241
H2	A2	0.241
H1	F1	0.241
H1	A1	0.241
H1	A2	0.241
H2	A3	0.243
H1	A3	0.243
H2	D3	0.245
H1	D3	0.248
H2	C8	0.262
H1	C8	0.262

H2	C6	0.266
H1	C6	0.266
H2	C1	0.277
H2	C2	0.277
H1	C1	0.277
H1	C2	0.277
G1	H1	0.328
G1	H2	0.330
G3	H1	0.335
G3	H2	0.337
B2	H1	0.342
B1	H1	0.342
B5	H1	0.342
B2	H2	0.346
B1	H2	0.346
B5	H2	0.346
B1	F2	0.348
B1	F1	0.349
B1	F3	0.349
B5	F2	0.349
B1	A3	0.349
B5	F1	0.351
B5	F3	0.351
B2	F2	0.351
B5	A3	0.351
B2	F1	0.353
B2	F3	0.353
B1	A1	0.353
B1	A2	0.353
B2	A3	0.353
B5	A1	0.355
B5	A2	0.355
B2	A1	0.356
B2	A2	0.356
B1	D1	0.356
B1	D2	0.356
B5	D1	0.358
B5	D2	0.358
B2	D1	0.360
B2	D2	0.360
B5	C8	0.363
B1	D3	0.363
B2	C6	0.365
B1	C6	0.365
B1	C8	0.365
G1	C8	0.365
B5	D3	0.365
G1	A1	0.367
G1	A2	0.367
B2	D3	0.367
B5	C6	0.369
B2	C8	0.369
G3	C8	0.369

B1	C1	0.371
B1	C2	0.371
G1	F2	0.371
G1	A3	0.371
B5	C1	0.372
B5	C2	0.372
G1	F3	0.372
G3	A1	0.372
G3	A2	0.372
G1	C6	0.372
G1	F1	0.372
B2	C1	0.374
B2	C2	0.374
G1	D1	0.374
G1	D2	0.374
G1	C1	0.376
G1	C2	0.376
G3	C6	0.376
G3	F2	0.376
G3	A3	0.376
G3	F1	0.378
G3	F3	0.378
G1	D3	0.378
G3	C1	0.379
G3	C2	0.379
G3	D1	0.379
G3	D2	0.379
G3	D3	0.383
B5	G1	0.385
B5	G3	0.385
B2	G1	0.399
B1	G1	0.399
B2	G3	0.401
B1	G3	0.401
