# Supplementary material for: Identification of a Genomic Reservoir for New TRIM Genes in Primate Genomes
Source: PLoS Genet. 2011 Dec 1;7(12):e1002388. doi: 10.1371/journal.pgen.1002388 (PMC3228819; doi:10.1371/journal.pgen.1002388)
Supplement: Table S3 — Summary of codeml simulations conducted with PAML. (PDF) [file pgen.1002388.s009.pdf]

**Supplemental Table S3.** PAML analysis of human TRIM sequences: Base pairs 1-237

| TRIM [a] dataset | $\omega_0$ [b] | codon freq. [c] | *M1a-M2a* $2\Delta\ell$ [d] | p-value | *M7-M8* $2\Delta\ell$ [d] | p-value | *M8a-M8* $2\Delta\ell$ [d] | p-value | tree length [e] | dN/dS (%) [f] | AA Positions of dN/dS>1 [g] * p>0.95 ** p>0.99 |
|---|---|---|---|---|---|---|---|---|---|---|---|
| TREE 3 | 0.4 | f61 | 10.6 | p = 0.005 | 17.1 | p = 0.0002 | 10.9 | p = 0.001 | 5.14 | 2.7 (27%) | I46*, P47*, F48*, V50**, E54**, E60**, H69**, L76* |
| | 1.4 | f61 | 10.6 | p = 0.005 | 17.1 | p = 0.0002 | 10.9 | p = 0.001 | 5.14 | 2.7 (27%) | I46*, P47*, F48*, V50**, E54**, E60**, H69**, L76* |
| | 0.4 | f3x4 | 6.5 | p = 0.04 | 10.4 | p = 0.005 | 7.3 | p = 0.007 | 4.96 | 2.7 (17%) | V50*, E54*, E60*, H69* |
| | 1.4 | f3x4 | 6.5 | p = 0.04 | 10.4 | p = 0.005 | 7.3 | p = 0.007 | 4.96 | 2.7 (17%) | V50*, E54*, E60*, H69* |
| TREE 4 (node collapsed) | 0.4 | f61 | 10.0 | p = 0.007 | 15.4 | p = 0.0004 | 10.4 | p = 0.001 | 5.11 | 2.7 (25%) | F48*, V50**, E54**, E60**, H69** |
| | 1.4 | f61 | 10.0 | p = 0.007 | 15.4 | p = 0.0004 | 10.4 | p = 0.001 | 5.11 | 2.7 (25%) | F48*, V50**, E54**, E60**, H69** |
| | 0.4 | f3x4 | 6.1 | p = 0.047 | 9.3 | p = 0.01 | 6.9 | p = 0.009 | 4.94 | 2.7 (16%) | H69* |
| | 1.4 | f3x4 | 6.1 | p = 0.047 | 9.3 | p = 0.01 | 6.9 | p = 0.009 | 4.94 | 2.7 (16%) | H69* |

PAML analysis of human TRIM sequences: Base pairs 238-1356

| TRIM [a] dataset | $\omega_0$ [b] | codon freq. [c] | *M1a-M2a* $2\Delta\ell$ [d] | p-value | *M7-M8* $2\Delta\ell$ [d] | p-value | *M8a-M8* $2\Delta\ell$ [d] | p-value | tree length [e] | dN/dS (%) [f] | AA Positions of dN/dS>1 [g] * p>0.95 ** p>0.99 |
|---|---|---|---|---|---|---|---|---|---|---|---|
| TREE 1 | 0.4 | f61 | 16.8 | p = 0.0002 | 30.2 | p < 0.0001 | 10.9 | p = 0.001 | 4.96 | 2.2 (18%) | R166*, C167*, R222*, Y320*, A323* |
| | 1.4 | f61 | 16.8 | p = 0.0002 | 30.2 | p < 0.0001 | 18.7 | p < 0.0001 | 4.96 | 2.2 (18%) | R166*, C167*, R222*, Y320*, A323* |
| | 0.4 | f3x4 | 17.0 | p = 0.0002 | 29.4 | p < 0.0001 | 17.7 | p < 0.0001 | 4.77 | 2.1 (18%) | R166*, C167*, Y320*, A323* |
| | 1.4 | f3x4 | 17.0 | p = 0.0002 | 29.4 | p < 0.0001 | 17.7 | p < 0.0001 | 4.77 | 2.1 (18%) | R166*, C167*, Y320*, A323* |
| TREE 2 (node collapsed) | 0.4 | f61 | 15.8 | p = 0.0004 | 28.8 | p < 0.0001 | 17.9 | p < 0.0001 | 5.01 | 2.1 (19%) | R166*, C167*, R222*, Y320*, A323* |
| | 1.4 | f61 | 15.8 | p = 0.0004 | 28.8 | p < 0.0001 | 17.9 | p < 0.0001 | 5.01 | 2.1 (19%) | R166*, C167*, R222*, Y320*, A323* |
| | 0.4 | f3x4 | 16.3 | p = 0.0003 | 28.1 | p < 0.0001 | 17.1 | p < 0.0001 | 4.82 | 2.1 (18%) | R166*, C167*, Y320*, A323* |
| | 1.4 | f3x4 | 16.3 | p = 0.0003 | 28.1 | p < 0.0001 | 17.1 | p < 0.0001 | 4.82 | 2.1 (18%) | R166*, C167*, Y320*, A323* |

a Dataset consisted of the 15 aligned human sequences: B1, B2, B5, F1, F2, F3, A1, A2, C1, C2, C6, C8, H1, H2, G1

b Initial seed value for $\omega$ (dN/dS) used in the maximum likelihood simulation

c Model of codon usage frequency

d Twice the difference in the natural logs of the likelihoods ($\Delta\ell$ x 2) of the two models being compared. This value is used in a likelihood ratio test along with the degrees of freedom. In all cases (M1a-M2a), (M7-M8), (M8a-M8), a model that allows positive selection is compared to a null model. The p-value indicates the confidence with which the null model can be rejected.

e The tree length is the number of substitutions per site along all branches in the phylogeny. It is calculated as the sum of the branch lengths, and is a representation of total diversity in the dataset

f dN/dS value of the class of codons evolving under positive selection in M8, and the percent of codons assigned to that class.

g Amino acid positions identified in the class of codons evolving under positive selection in M8 with a posterior probability >0.95 by Naive Emperical Bayes (NEB). Coordinates correspond to the TRIM49(F1) protein.
