# Supplementary material for: Identification of a Genomic Reservoir for New TRIM Genes in Primate Genomes
Source: PLoS Genet. 2011 Dec 1;7(12):e1002388. doi: 10.1371/journal.pgen.1002388 (PMC3228819; doi:10.1371/journal.pgen.1002388)
Supplement: Table S4 — MLPA probe sequences. LPO and RPO stand for left probe oligo and right probe oligo, respectively. Green and blue letters indicate forward and reverse universal primer binding sites, respectively. Lower case letters represent stuffer sequences that are built into each probe pair so that it produces a PCR product of unique size. (PDF) [file pgen.1002388.s010.pdf]

Supplemental Table S4. MLPA probe sequences

#	Name[a]	Oligonucleotide sequence (5'-> 3')[b]	Primer length (bp)	Expected fragment size (bp)	Tm (°C)[c]	GC (%)[c]
1	5FR_LPO	GGGTTCCCTAAGGGTTGGAtgtgggaggcgaaaattggcATAAGCTCAAACTGTCACCCATGTCGTGACTTT	72		73.73	42
	5FR_RPO	AAACTCGTCTTCTACTCTACACCACAACACACACAacgtccggatgctgatgatggcaagcTCTAGATTGGATCTTGCTGGCAC	84	156	72.57	44
2	B3/4_LPO	GGGTTCCCTAAGGGTTGGAtaaaaaactaccgtCTAATGCCATCAGAAACATCTCGTAACAAGCA	65		72.84	41
	B3/4_RPO	GAAGGTGAGGGATCTGTGAACATGACCTAACCATGCgaaaagtcggtggaTCTAGATTGGATCTTGCTGGCAC	73	138	79.73	50
3	GAP1-1_LPO	GGGTTCCCTAAGGGTTGGAtcggcgttGATGAAGGTTCCTATATAAGATGATGGGGTAG	59		69.69	41
	GAP1-1_RPO	AGGAGCAGGACTTAGAATAGTGGAGACTGGCCTTAAACTGACttccggaaTCTAGATTGGATCTTGCTGGCAC	73	132	78.51	48
4	GAP1-2_LPO	GGGTTCCCTAAGGGTTGGAtgtgggaggcgaaaattggcACCGGAGGAGGGCGTCCTGGAGGCGCGGGGCAGA	73		93.33	76
	GAP1-2_RPO	GGCGGACGCTGGTGCGGTGTGGGGTGAGCTTgaaaagtcggtggaTCTAGATTGGATCTTGCTGGCAC	68	141	89.14	71
5	GAP1-3_LPO	GGGTTCCCTAAGGGTTGGAtcagcgcaacacAACCTCATGTGGACCTCATGGAATTTCCAAA	62		74.36	42
	GAP1-3_RPO	ACATGGGGCCTTGGCATTGTGGCACCTTCCTGCTACACccttatctggttTCTAGATTGGATCTTGCTGGCAC	73	135	85.77	58
6	F1/2_LPO	GGGTTCCCTAAGGGTTGGAtgcaggtcgaaaatgggtggatggcACTGAGTAGGTTTTTCATGGTTACCACAGAC	75		70.05	42
	F1/2_RPO	CATAGACCTTCTGGGCCTCTTCTCCCTTCACTTCTaggaaagcaatactctgggacacgtaTCTAGATTGGATCTTGCTGGCAC	84	159	78.9	51
7	GAP2_LPO	GGGTTCCCTAAGGGTTGGAtgtgaatgggGTTTCCTGTATGGGATGGATTCAGGGATCAATT	62		75.28	42
	GAP2_RPO	ACATGTCGTCAACTCCATAAATCCTCAAATCTCTCAgcgagTCTAGATTGGATCTTGCTGGCAC	64	126	73.99	39
8	E1/2_LPO	GGGTTCCCTAAGGGTTGGAtgcaggtcgaaaaatgggtggatggcCGCTGGGAGGAAGGCCAAGCTCCAA	70		80.48	64
	E1/2_RPO	AGGGCTGTCCTGTGTGTGGGAAAATCCCCCAGAAaggaaagcaatactctgggacacgtaTCTAGATTGGATCTTGCTGGCAC	83	153	83.24	56
9	GAP3_LPO	GGGTTCCCTAAGGGTTGGAtaggccGAGGAACTCTAGGAGCCAGAAAACCT	51		76.76	48
	GAP3_RPO	CACAGCCTTCTCCTCCAGCAGGGTTAGATTGGGCAATTagcgaTCTAGATTGGATCTTGCTGGCAC	66	117	88.58	61
10	D1/2_LPO	GGGTTCCCTAAGGGTTGGACTGCAGGATTATGTGAATTTAAGGCTAGAAGCTATG	55		72.24	39
	D1/2_RPO	AGAGCTGAGTATCAGAAGATGGCTGCATTTCACCATTCTAGATTGGATCTTGCTGGCAC	59	114	76.28	44
11	C1/2-1_LPO	GGGTTCCCTAAGGGTTGGAATGCTGGAAGGTTAGTACCGTATTACTCTACCTTCTG	56		72.73	43
	C1/2-1_RPO	CAGGAACTTATAGTGAACAAATGGGTGACTCTTCTAGATTGGATCTTGCTGGCAC	55	111	71.11	41
12	C1/2-2_LPO	GGGTTCCCTAAGGGTTGGAtcggtgagacgtgggaggcgaaaattggcgAATGTACCATAAAGCAGATGTGGAGCTACTCCT	82		71.59	42
	C1/2-2_RPO	GGTACGGACTGACCATGGGGTATCATGATtgaccgtgtggcttacctgaccgccggtatcTCTAGATTGGATCTTGCTGGCAC	83	165	75.59	52
13	GAP5_LPO	GGGTTCCCTAAGGGTTGGACATTTCAAATTCGTGGTCTTTGTTAGAGCAC	50		71.48	39
	GAP5_RPO	ATAAGCCGGTGCGCTAGGAGATGTAGTCTAGATTGGATCTTGCTGGCAC	49	99	72.93	50
14	B1/2_LPO	GGGTTCCCTAAGGGTTGGAtattacgcccgtgccttatccggagaggATACGCAGGTGATATTTGCATGTCCTGGCAGCAC	81		78.56	50
	B1/2_RPO	TGTCCAGCAAAGGCTTCCTGTCTCTGAGGATaggtaaagcaatactctgggacacgtaTCTAGATTGGATCTTGCTGGCAC	81	162	77.31	52
15	GAP6-1_LPO	GGGTTCCCTAAGGGTTGGAAGGCCTAGGTGGCTTGACCGGTCAA	44		76.31	60
	GAP6-1_RPO	GCTGAGAAGTTCGACACCAGGCTAGTCTAGATTGGATCTTGCTGGCAC	48	92	72.26	56
16	GAP6-2_LPO	GGGTTCCCTAAGGGTTGGAGCTGGCTTGAGCTCTCTCCATAC	42		69.79	57
	GAP6-2_RPO	TCAGGCTGTGATGCAGGAGCATGTGGAGAATCTAGATTGGATCTTGCTGGCAC	53	95	78.87	53
17	A1/2_LPO	GGGTTCCCTAAGGGTTGGAGGTGTGTTAAGAATGACATTCAGTGCAGC	48		72.15	45
	A1/2_RPO	CTCTTTACCACCTCCCCACTTACACTGCAGTATGTCTAGATTGGATCTTGCTGGCAC	57	105	76.55	50
18	M-Uniq_LPO	GGGTTCCCTAAGGGTTGGAtcatccggtgaagagattTTGGCATTGTCATGTCCCACTGGTCGGCAGCTGAGG	73		85.81	58
	M-Uniq_RPO	GTCCTTCTGCTCTAACCTGAGAATAGACAAAACAAtgctgcgtgtggatgaTCTAGATTGGATCTTGCTGGCAC	74	147	72.79	40

[a] LPO and RPO stand for left probe oligo and right probe oligo, respectively.

[b] Green and blue letters indicate forward and reverse universal primer binding sites, respectively. Lower case letters represent stuffer sequences.

[c] Tm and GC content are estimated by Oligo Analysis Tool (http://www.operon.com/technical/toolkit.aspx).
