# Supplementary material for: Identification of a Genomic Reservoir for New TRIM Genes in Primate Genomes
Source: PLoS Genet. 2011 Dec 1;7(12):e1002388. doi: 10.1371/journal.pgen.1002388 (PMC3228819; doi:10.1371/journal.pgen.1002388)
Supplement: Table S6 — Human individuals surveyed in the MLPA analysis. All genomic DNA samples were obtained from Coriell except where indicated. (PDF) [file pgen.1002388.s012.pdf]

Supplemental Table S6. Human samples used in the MLPA assay.

#	Catalog ID	Race or description	Sex
1	NA10851	Caucasian (CEPH/UTAH)	Male
2	NA10861	Caucasian (CEPH/UTAH)	Female
3	NA12740	Caucasian (CEPH/UTAH)	Female
4	NA15755	Caucasian (Icelandic)	Male
5	NA17315	South America	Male
6	NA17301	South America	Male
7	NA17378	Africans North of the Sahara	Male
8	NA17341	Africans South of the Sahara	Female
9	NA17349	Africans South of the Sahara	Male
10	NA17107	African American	Male
11	NA17396	Mexican Indian	Male
12	NA18502	Yoruba in Ibadan, Nigeria	Female
13	NA18573	Han Chinese in Beijing	Female
14	NA18563	Han Chinese in Beijing	Male
15	NA18969	Japanese in Tokyo	Female
16	NA18975	Japanese in Tokyo	Female
17	NA18997	Japanese in Tokyo	Female
18	K7 [a]	Korean	Female
**HD25 Aboriginal Tribe from Taiwan (Ami)**			
19	NA13607	Ami population	Male
20	NA13608	Ami population	Male
21	NA13609	Ami population	Male
22	NA13610	Ami population	Male
23	NA13611	Ami population	Male
24	NA13612	Ami population	Male
25	NA13613	Ami population	Male
26	NA13614	Ami population	Male
27	NA13615	Ami population	Male
28	NA13616	Ami population	Male
**HD32 Chinese**			
29	NA16654	Chinese	Male
30	NA16688	Chinese	Female
31	NA16689	Chinese	Male
32	NA17014	Chinese	Male
33	NA17015	Chinese	Male
34	NA17016	Chinese	Male
35	NA17017	Chinese	Male
36	NA17018	Chinese	Female
37	NA17019	Chinese	Female
38	NA17020	Chinese	Male
**HD13 Southeast Asians (Excluding Japanese and chinese)**			
39	NA17081	Korean	Female
40	NA17082	Vienamese	Male
41	NA17083	Vienamese	Female
42	NA17084	Filipino	Female

43	NA17085	Filipino	Male
44	NA17086	Cambodian	Female
45	NA17087	Cambodian	Female
46	NA17088	Cambodian	Male
47	NA17089	Filipino	Male
48	NA17090	Korean	Male
**HD24 Han people of Los angeles**			
49	NA17733	Chinese	Female
50	NA17734	Chinese	Female
51	NA17735	Chinese	Female
52	NA17736	Chinese	Male
53	NA17737	Chinese	Male
54	NA17738	Chinese	Female
55	NA17739	Chinese	Female
56	NA17740	Chinese	Female
57	NA17741	Chinese	Female
58	NA17742	Chinese	Male
59	NA17743	Chinese	Male
60	NA17744	Chinese	Female
61	NA17745	Chinese	Female
62	NA17746	Chinese	Female
63	NA17747	Chinese	Female
64	NA17749	Chinese	Male
65	NA17752	Chinese	Female
66	NA17753	Chinese	Male
67	NA17754	Chinese	Female
68	NA17755	Chinese	Male
69	NA17756	Chinese	Female
70	NA17757	Chinese	Female
71	NA17759	Chinese	Male
72	NA17761	Chinese	Male

[a] DNA sample from Dr. Heui-Soo Kim, Pusan National University, Busan 609-735, Korea
