# Supplementary material for: Identification of a Genomic Reservoir for New TRIM Genes in Primate Genomes
Source: PLoS Genet. 2011 Dec 1;7(12):e1002388. doi: 10.1371/journal.pgen.1002388 (PMC3228819; doi:10.1371/journal.pgen.1002388)
Supplement: Table S7 — Primers used in this study. Primers are shown for the amplification of TRIM transcripts from cDNA. Also shown are primers used to amplify full-length genes for viral restriction assays. (PDF) [file pgen.1002388.s013.pdf]

Supplemental Table S7. Primers used in this study

	Locus	Forward (5' -> 3')	Reverse (5' -> 3')	A.T.[a] (°C)
**Primers used for the amplification of spliced partial transcripts of *TRIM* genes**				
1	G1	ATCCCCAAGGCATGCTACTCAC	AATGAGGGAACTTTGGGCAACA	59
2	G1'	ATCCCCAAGGCATGCTACTCAC	AATGAGGGAACTTTGGGCAACA	59
3	B3, B4	ATTCTGATGCCCTGCGAGT	TGTTGGGTCATTCTCACTTGAC	59
4	F1, F2	CAGTCTCTGGCTATTCCTGAGC	AGTAATATTTGCCCGAGGTGAA	59
5	E1, E2	GACCTCACAACATCAACAGCTC	CACATCCTCAGAAAGGCTCATA	59
6	D1, D2, D3	CACTCACAGGGAGACAAAGAAG	TCTTCTTCATGGTGAAATGCAG	60
7	C3, C6	CCAGGGAGCTTTTAAGAGGAAT	TCCCTCCTCTCCATCTATCTTG	59
8	B1, B3, B4	CTGCAAGTCTTCCAGAATGAGC	GGAGGTCACAATAAATCATCGTGAAACC	59
9	A1, A2	AGGAGGGAGATTTTAAGAGGAAC	CGTCGGAAGATATGACTGTTGG	59
10	F3	GTTTCTACCTCAACTGGaAAGACAG	TCCCTCCTATGAGCCATTTTG	59
11	B1, B6	TATTTCTCGATGAGGAGGAGCA	GGAGGAAGGAGGAAAACCATAG	59
**Primers used to amplify full length *TRIM* genes for functional studies**				
1	B1	CTGCAAGTCTTCCAGAATGAGC	GGAGGTCACAATAAATCATCGTGAAACC	59
2	F1, F2, F3	GGAATCTTACAGGTCTTTCAGGGG	TGTGAACACATTTCTGATTTGTCTCTGG	59
3	A1, A2	ATGAATTCTGGAATCTCGCAAGTCTTCC	TCAGAGGTGAACACAGCAAAAGATAGG	58
4	B5[b]	TTCCTGTCCAGCCCTACCCCAG	GGGAGCTCACAATAAATCATCATGAAACC	63.5
5	B5-SOE[b]	GTGGATAATGCTCTGAGTACAGAA	TCATGTACAACCAAAGCAAAAGAAAGGCC	59
6	Rhesus TRIM5α-SOE[b]	GCCACCATGGCTTCTGGAATCC	TTCTGTACTCAGAGCATTATCCACCCAGTAGCGTCGGGCA	59

[a]A.T. stands for annealing temperature.

[b] A chimeric B5 *TRIM* gene was constructed: rhesus TRIM5α (excluding final exon encoding B30.2) + the B5 B30.2 amplified from gDNA

SOE = Spliced Overlap Extension (for joining pieces)
